# Collaborative Learning Activity Utilizing Evidence-Based Medicine to Improve Medical Student Learning of the Lifestyle Management of Obesity

**DOI:** 10.15766/mep_2374-8265.10426

**Published:** 2016-07-21

**Authors:** Magdalena Pasarica, David M. Harris, Judith Simms-Cendan, A. Laurel Gorman

**Affiliations:** 1 Associate Professor of Medicine, University of Central Florida College of Medicine; 2 Associate Professor of Physiology, University of Central Florida College of Medicine; 3 Associate Professor of Obstetrics and Gynecology, University of Central Florida College of Medicine; 4 Vice President of the Faculty Council, University of Central Florida College of Medicine; 5 Associate Professor of Pharmacology, University of Central Florida College of Medicine; 6 Associate Professor of and Medical Education, University of Central Florida College of Medicine

**Keywords:** Evidence-Based Medicine, Active Learning, Nutrition, Obesity, Problem-Based Learning, Exercise, Collaborative, Life Style, Group

## Abstract

**Introduction:**

Innovative and effective curricula for medical students and physicians are needed to increase knowledge and confidence for instructing patients on lifestyle management of diseases. We developed an active collaborative session that integrates evidence‐based medicine (EBM), clinical decision-making, nutrition, exercise, and personalized patient care for the instruction of lifestyle management of obesity in the preclinical medical curriculum.

**Methods:**

Before the session, learners critically appraised an EBM article (meta‐analysis of commercial weight-loss programs' efficacy). In class, there was an EBM discussion assessed and facilitated by multiple-choice questions, followed by a collaborative activity where learners solved a clinical scenario of a patient who wants to use a commercial weight-loss program. Each small group was assigned to a different program but given the same clinical scenario. The objectives of the session were to identify and interpret EBM/non‐EBM resources in order to describe the components, advantages, and disadvantages of the weight-loss programs, make a personalized clinical recommendation, and present it to the class.

**Results:**

Generating debate and fostering engagement, the session was perceived as a positive learning experience by the learners. By accomplishing the learning objectives, the participants became well versed in various weight-loss programs.

**Discussion:**

Our results suggest that learners developed interpretation and knowledge integration skills, which may increase their comfort in discussing the lifestyle management of obesity and other diseases. This activity is designed to be implemented at other institutions seeking to integrate active collaborative learning of nutrition, exercise, and clinical decision-making during preclinical and clinical medical education and clinical practice.

## Educational Objectives

By the end of this session, learners should be able to:

1. Critically appraise evidence-based medicine (EBM) and non?EBM resources related to popular commercial weight-loss programs.
2. Describe the components, advantages, and disadvantages of popular commercial weight-loss programs.
3. Develop a personalized recommendation for a patient who wants to use a commercial weight-loss program for the management of obesity.

## Introduction

The need for innovative and effective practical lifestyle management curricula for medical students has been recognized as essential and timely by leading governmental and academic organizations, including the National Institute of Diabetes and Digestive and Kidney Disease, the National Academy of Science, Harvard Medical School, and the Lifestyle Medicine Think Tank.^1–4^ Obesity is an epidemic in the United States and worldwide. Importantly, in obese individuals, weight loss improves the control of comorbidities associated with obesity, including type 2 diabetes, hypertension, and hyperlipidemia. Most adult Americans recognize the need to lose weight; however, many physicians are uncomfortable prescribing lifestyle management of obesity.^5^ This has led to a suboptimal treatment of the obesity epidemic and associated diseases.

There is a need to teach nutrition to medical students in an innovative way that promotes critical thinking in order to maximize retention and stimulate interest.^3^ The United States Medical Licensing Examination (USMLE) Step 1 exam assesses for nutrition knowledge.^6^ Several institutions have developed recommendations for medical school nutrition curricula,^7^ surveys to evaluate patients' nutrition status,^8^ online self-learning modules,^9^ lectures, and team-based learning activities for nutrition education.^10–14^ However, to our knowledge, none of these resources have managed to integrate evidence-based medicine (EBM), clinical decision-making, nutrition, exercise, and personalized patient care in a collaborative active learning session. We developed a session for instruction in the lifestyle management of obesity utilizing all of the aforementioned techniques/competencies that permitted collaborative and peer education and allowed instruction on multiple topics in a relatively short amount of time in the curriculum. At the end of the session, learners should be able to develop a personalized recommendation for a patient who wants to use a commercial weight-loss program for the management of obesity. This will move the learners higher on Miller's pyramid of clinical competence,^15^ up to the Shows How level. The overall goal of this activity is for the learners to develop searching, interpretation, and knowledge integration skills that may increase their confidence in making recommendations for the lifestyle management of obesity and other diseases. We recommend its use in the education of preclinical and clinical medical students, medical residents, and fellows, as well as physicians who have a basic knowledge of the lifestyle management of obesity and the effect of weight loss on associated diseases. A similar approach could be used in teaching lifestyle management of other chronic diseases.

This session was developed after a similar clinical scenario that a medical student encountered in the clinical setting. The student felt that the current class curriculum was largely theoretical and insufficient to make a practical personalized lifestyle recommendation. Several other students at our institution requested more practical nutrition teaching, which supported the need for the development of a practical session. Magdalena Pasarica, a medical educator with expertise in lifestyle management of obesity (family medicine physician with a PhD in nutrition), developed this session in collaboration with David M. Harris, Judith Simms-Cendan, and A. Laurel Gorman, experts in medical education, active learning, and EBM.

## Methods

This session was mandatory for University of Central Florida College of Medicine second-year medical students as part of their preclinical training and incorporated collaborative learning of several skills (Figure 1).

The prerequisites, presession task, and active session tasks with their respective time allotments are briefly presented in a flowchart (Figure 2) and are detailed in the text that follows.

Prior to the session, learners received a 1-hour lecture on the lifestyle management of obesity with the following objectives: to define obesity, its prevalence, and related comorbidities and to discuss obesity management guidelines. Students had some background knowledge of EBM, useful online search methods, biostatistics, and clinical study design as part of the EBM longitudinal curriculum.

The presession task consisted of reading an EBM article^16^ and completing an individual critical appraisal using an EBM interpretation guide (as detailed in Appendix A). The EBM article was a recent meta‐analysis of popular commercial weight-loss programs, with a focus on their efficacy and potential harms, accompanied by a brief description of each program. The EBM article was free to access, had been published in a reputable medical journal, and was selected because it presented a recent overview of lifestyle management of obesity programs; as such, it was well suited as a resource for the active session.

In part 1 of the active session, students used an audience response system (ARS) to answer five EBM questions (Appendix A) designed to facilitate discussion about the appraisal of the EBM article assigned. The students were not allowed to utilize any outside resources (including books and the Internet). Questions were answered on an individual basis. Immediate feedback was provided after each question by the instructor.

In part 2 of the active session, students worked in groups to solve a clinical scenario using the EBM article and other EBM/non‐EBM articles as needed (see Appendix A for the student instructions and clinical scenario). In this frequently encountered scenario, a patient was instructed to lose weight and came to the clinic because she desired to use a commercial weight-loss program. The patient asked for recommendations based on the program components, the advantages and disadvantages of the program, and the suitability of this program for her health. Each group was randomly assigned to one particular commercial weight-loss program as discussed in the EBM article, but within the same clinical scenario. Students were permitted to access the Internet and were encouraged to appraise the evidence from other EBM articles using a similar approach found in the EBM guide (Appendix A). Teams were instructed to record their recommendations on a portable whiteboard (see Appendix B for team boards). Each team designated one member to present the team's work in front of the class. The other students were present in front of the class to answer any questions asked by their colleagues. Students were encouraged to debate the recommendations with the presenters. Learners received immediate formative feedback for their performance.

The entire activity was mandatory but was formatively assessed; it did not count towards students' grades in the endocrine module. Students were encouraged by the instructor to prepare well in order to derive the greatest benefit from the session.

Our session was moderated by an instructor with expertise in nutrition, clinical experience in treating obese patients, and extensive knowledge of the EBM article. The session was coordinated by two staff members (see Appendix B for instructions to the session coordinator for tasks before and during the session). A PowerPoint presentation was used to deliver the instructions and project the clinical case. An ARS was employed to deliver the multiple-choice questions. In order for all students to see the whiteboards, a camera was used to display them on a projector screen.

Student perception was analyzed by evaluating comments pertaining to this session from a deidentified overall postmodule survey assessing the instructor's performance within the course that incorporated this activity. Two open-response questions were relevant: What are this faculty member's teaching strengths? What suggestion do you have for this faculty member? Only the comments pertaining to this particular session are summarized in the Results section. This study was approved by the University of Central Florida's Institutional Review Board.

## Results

Our activity was used once as a mandatory session for second-year medical students (112 students) in the endocrine module and as part of the nutrition longitudinal curriculum.

During part 1 of the active session, students answered five EBM questions (see Appendix A for questions and answers). The first question regarded the main purpose of the EBM article. Only 2% of students answered it correctly (the study compared harms of the programs, in addition to efficacy for weight loss). Most students answered that the study compared health benefits (which was incorrect and created the most debate). However, the study compared the efficacy of the weight loss but not the effects on other diseases associated with obesity, such as hypertension and diabetes mellitus type 2. The take-home message was that careful reading of the purpose of a study is essential for a critical appraisal. The second question concerned the comparison group. Most students (96%) correctly indicated behavior counseling. This question did not generate any debate. The take-home message was that knowing the characteristics of the comparison group is essential when interpreting the effect of an intervention. The third question dealt with the weight-loss difference between the most effective program and the comparison program. Most students (71%) correctly indicated 2.6%. This question generated some debate, as students felt that the question was too detailed. However, the other answers differed greatly from the correct answer, and the program in question was the most efficacious according to the meta‐analysis. The take-home message here was to look at the effect size always, not only when the difference is significant. The fourth question asked about the source of funding. Most students (86%) correctly indicated none. This question did not generate any debate. The take-home message was that one should always look for the source of funding when appreciating the validity of the conclusions. The fifth question regarded the main limitation of the study. Results were mixed. Most students (40%) answered that the main limitation was not consistent blinding. The other answers (only US patients, no comparison between the commercial programs, only focused on weight loss) were each chosen by approximately 20% of students. This question generated a useful critical-thinking debate and a review of proper study design and study limitations.

Students reviewed the efficacy, safety, and side effects of the program assigned using EBM data gathered by searching within PubMed. They discussed the patients' experience and satisfaction with the weight-loss program using non‐EBM data from the program websites and patient discussion websites. There was an active debate between the team members to decide what to consider an advantage and/or disadvantage as well as how to develop the personalized patient recommendation.

Group-written answers to the clinical scenario are presented in Appendix B. When questioned, the group representative verbally provided additional significant information. Each group presented examples of several meals, snacks, and exercises included in each of the weight-loss programs. They presented the advantages and disadvantages of the weight-loss program from the medical point of view and from the patient's perspective. The groups integrated elements of the social history, such as patient income or work schedule, in their recommendations. For example, programs that provided cooked meals were recommended to patients too busy to cook. Programs that were low cost were recommended to patients with low income. Therefore, students performed a personalized patient recommendation. When evaluating the session, students had mostly positive comments and gave recommendations for improvement. Students appreciated the session because they perceived it as active, engaging, making nutrition fun, better for learning, and informative. Some students would have preferred to learn similar material by attending a lecture, and some preferred to have an activity/lecture focusing on other topics more closely related to USMLE Step 1 material. Some students recommended working in smaller groups.

## Discussion

This session addressed the timely and relevant topic of lifestyle management of obesity and integrated EBM, clinical decision-making, nutrition, exercise, and personalized patient care. This was accomplished by using 1 hour of lecture and the described active learning session in only 2 hours of time in the curriculum. This session promoted active collaborative learning for the development of an educated clinical recommendation and therefore moved the learners higher on Miller's pyramid of clinical competence, to the Shows How level, signifying higher learning impact. Our findings indicate that our learning objectives were achieved and suggest that learners completing this activity may develop interpretation and knowledge integration skills that may increase their confidence in discussing lifestyle management with patients. During the active session, the instructor perceived the students as being engaged and having a positive attitude.

As more medical institutions incorporate active collaborative learning, we recommend that this session be used to instruct the lifestyle management of obesity across the preclinical and clinical education continuum, as well as to meet the continuing education needs of practicing physicians. This session can be implemented as is (using the faculty step-by-step guide and instructions in Appendix B and student material in Appendix A) or utilized as a template for the instruction of lifestyle management of other diseases. For example, in our institution, we successfully used this template to instruct on the lifestyle management for the prevention of cardiovascular disease. Depending on the number of students, the active session should last 1‐2 hours. Learners should have previous knowledge of biostatistics, clinical research methodology, and lifestyle management of obesity. The lecture on obesity can be obtained using one of the multiple resources available online and through MedEdPORTAL.^9,10,13,14^ For the preclass assignment, the meta‐analysis cited here can be used^16^; we recommend updating the reference as newer research publications emerge. Instructors should have knowledge of the lifestyle management of obesity and currently available weight-loss programs. Two staff members should be present to facilitate the session. A computer with PowerPoint capabilities and ARS technology and a camera connected to the projector are recommended for this session. The multiple-choice questions, instructions, and clinical scenario may be delivered as paper handouts.

Based on our experience and the learners' evaluations, we plan to make a few minor revisions for improvement as follows: The groups will be smaller (maximum of six students to increase collaborative learning), students will report all the resources that they used in addition to the board presentation, and at least 5 minutes will be spent before the session discussing the importance of this delivery method. This can be achieved by mentioning the long-term goal of this activity, which is for students to develop searching, interpretation, and knowledge integration skills that will be useful in clinical practice when making recommendations for the lifestyle management of obesity and other chronic diseases; the fact that nutrition questions will be on their USMLE Step 1 exam; and the importance of active versus passive learning.

This session had several challenges. It required 2‐3 hours of curriculum time, which may be perceived as excessive if the significance of the session is not adequately described to module/course directors. Learners who are used to lectures only as an educational modality may perceive this activity as cumbersome, which may be overcome by explaining the benefits of active learning and why the pedagogy was chosen. To make it more engaging, a short video could be filmed to bring the material to life. A limitation of this session is that the survey deployed evaluated the overall instructor's performance in the endocrine module, which may have limited the response rate. For programs that would like to get more representative data on student perception, a survey specifically dedicated to this session should be designed.

## Appendices

A. Student Presentation.pptx
B. Facilitators Guide.docx All appendices are peer reviewed as integral parts of the Original Publication.
