# Supplementary material for: Collaborative Learning Activity Utilizing Evidence-Based Medicine to Improve Medical Student Learning of the Lifestyle Management of Obesity
Source: MedEdPORTAL. 2016 Jul 21;12:10426. doi: 10.15766/mep_2374-8265.10426 (PMC6464419; doi:10.15766/mep_2374-8265.10426)

Appendix B​: Faculty guide

1. Instructions for faculty development
2. Instructions for session coordinator
3. Step-by-step recommendations for instructors planning to implement this activity
4. White boards with team recommendations from our session
5. Instruction for faculty development

For part 1 of the active session, take the opportunity to discuss EBM principles applied to the assigned reading. If the majority of class answered correctly (>70%), you may not need to discuss the correct options. For the fifth question, guide the students to discuss why all of those limitations are correct and important

For part 2 of the active session, take your time presenting the case and the instructions. Leave the clinical scenario on the board so that students can refer to it if they need to during the activity. When the team activity starts, walk around the groups and interact with students as they have questions, concerns or remarks. Guide them but do not give them the correct answer. Allow them to develop their opinion.  During the group presentation, ask at least one pertinent question per group and wrap up with a “take home message” about each weight-loss program. Praise students for using ample EBM/non‐EBM resources and considering the characteristics of the patient in their recommendation. Encourage debate and discussion among learners by asking open ended questions.

1. Instructions for session coordinator

One week before the session, the coordinator sent the pre‐session task, the link to the EBM article​^15^ and sessions’ objectives to the students. Students were assigned to 10 groups (8‐12 students/group). The following items were supplied to the classroom: a) 10 clean whiteboards (32”/42”) with 4 markers of different colors; b) 10 flags with group numbers arranged in consecutive order and instructions for students to leave the flags in their assigned spots and sit with their team; c) the list with group student assignments; and d) support for the white boards at a location that is compatible with the camera in front of the class.

During the active session, the coordinator guided students for the assignment time flow, asked teams to be ready to present before the other team completed their presentation, timed the presentations and flag students when 30 seconds remained, arranged the camera to project the larger view of the students’ white boards and handed out microphones to the presenting students.

1. Step-by-step recommendations for instructors planning to implement this activity

Step 1: discuss this opportunity with the course/module director to get their approval for running this session. Use Figure 1 and 2 and session objectives to enhance the presentation. Once the session is approved, more to step 2.

Step 2: work with the session coordinator to have all your resources/technology available. Use the instructions for session coordinator from Appendix B.

Step 3: customize the design of your session depending on the number of students and time allowed in the curriculum ( you may need to have more or less programs assigned for the teams, or you may have to only allow a selection of teams to present their findings)

Step 4: make sure you have enough staff members helping you (especially if you have a class larger than 40)

Step 5: If you are planning to deploy this more than 4 years after this manuscript was published, please do a quick pubmed search to identify the need for replacement of the EBM article

Step 6: communicate with the session coordinator to communicate with the students about the requirement of EBM critical review at least 1 week before your session

Step 7: get familiar with the assigned EBM article

Step 8: put the materials found in Appendix A in a power point, and you are ready for your session! Enjoy!

1. White boards with team recommendations from our session


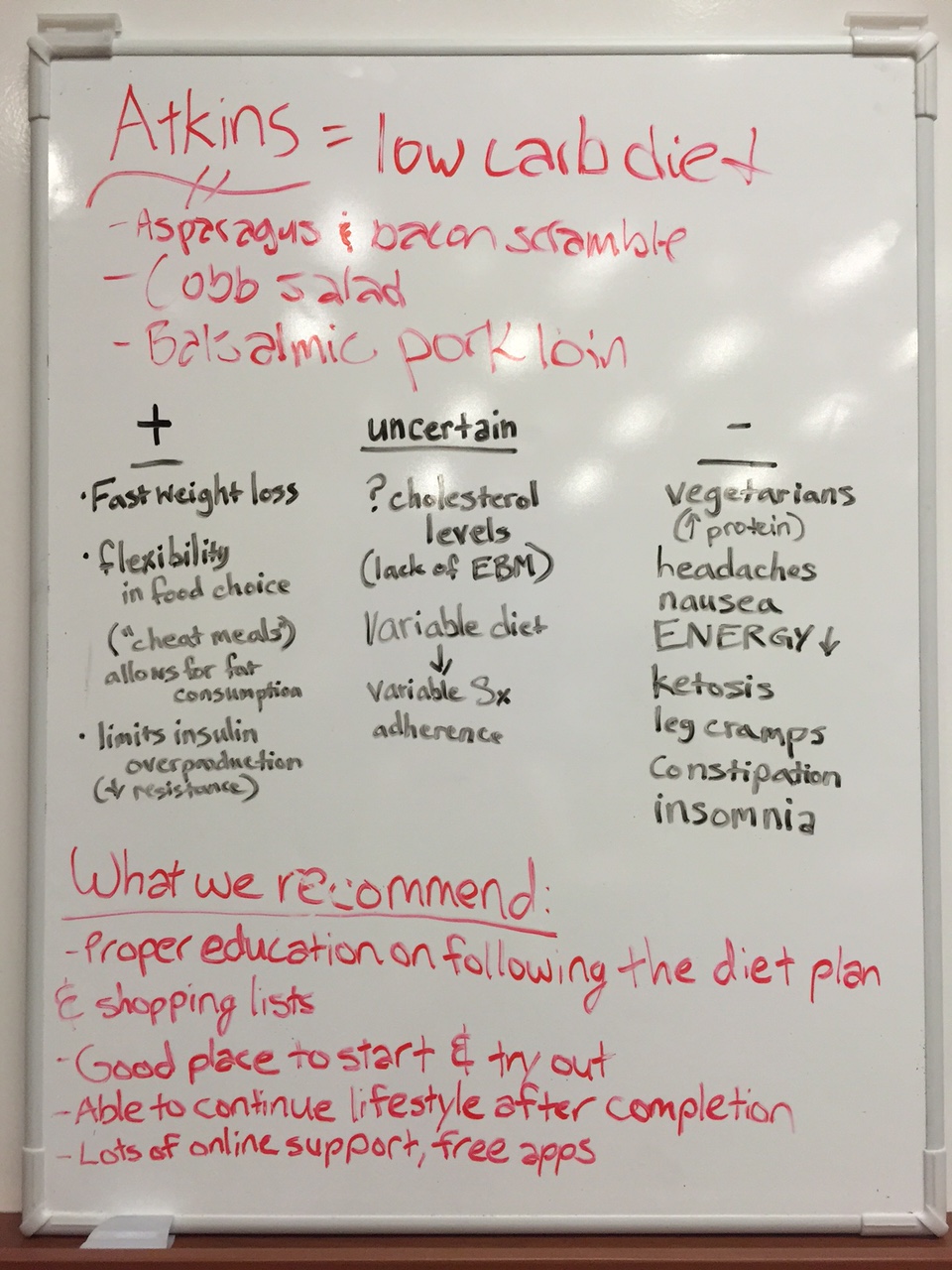


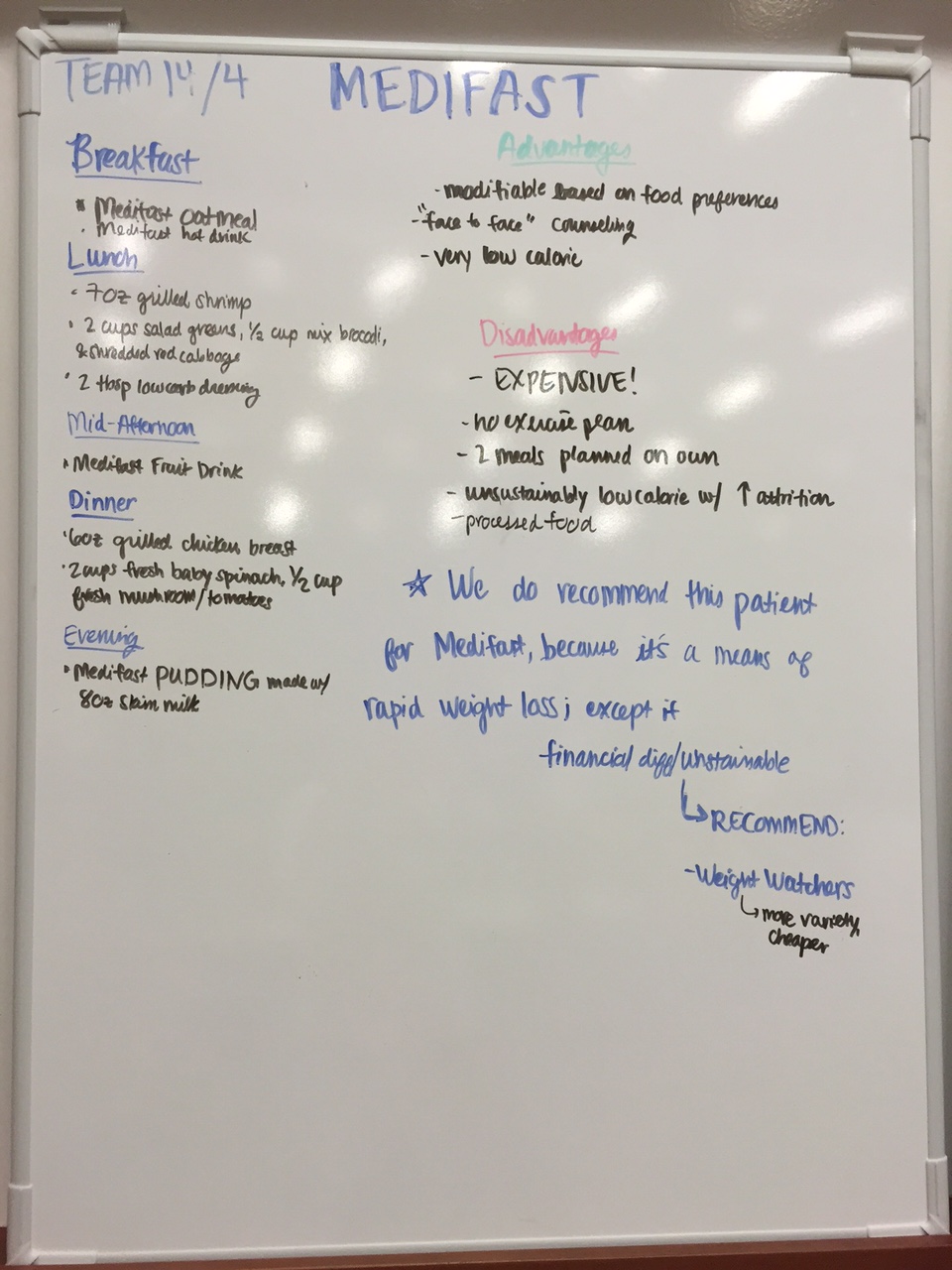


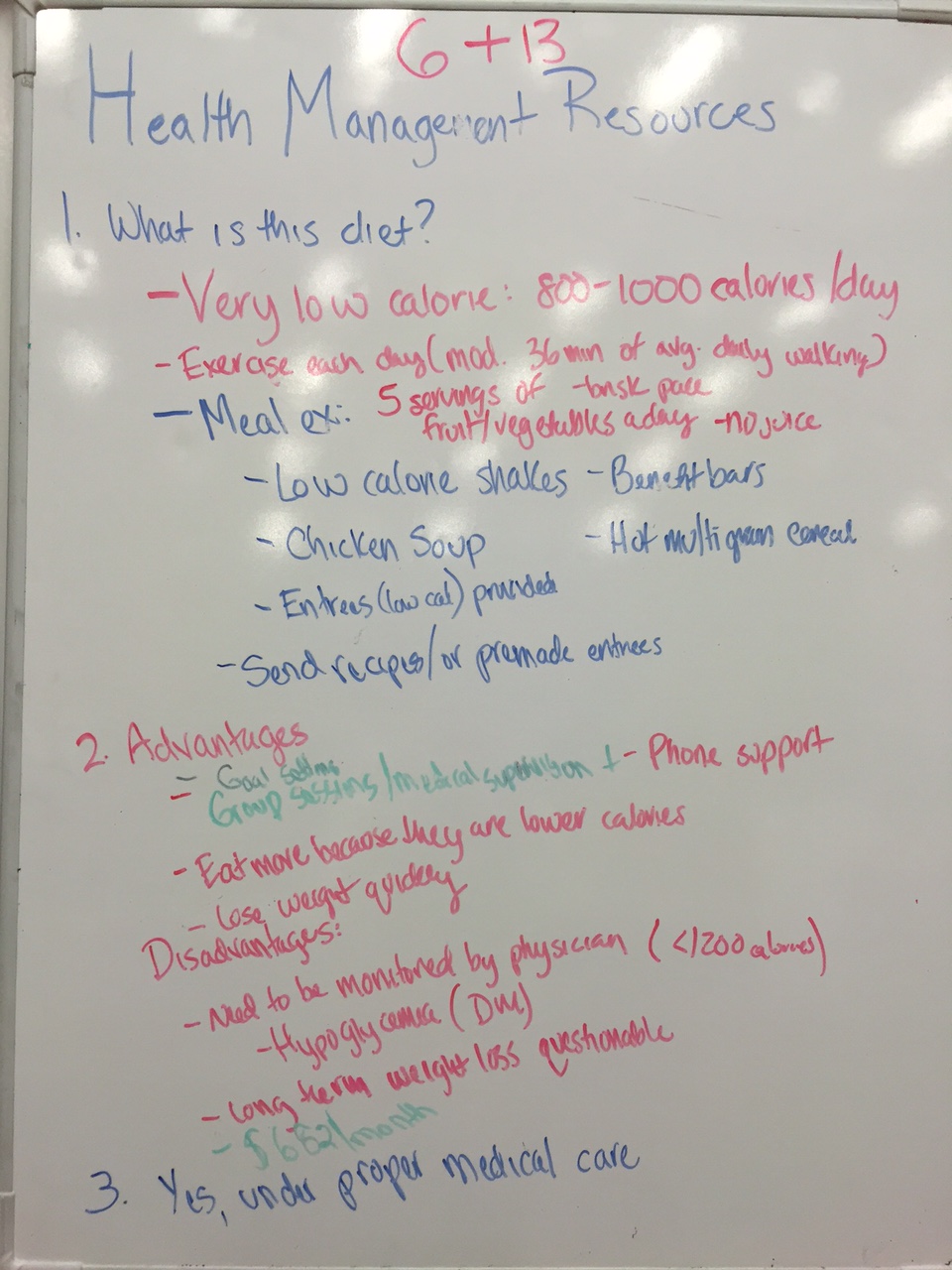


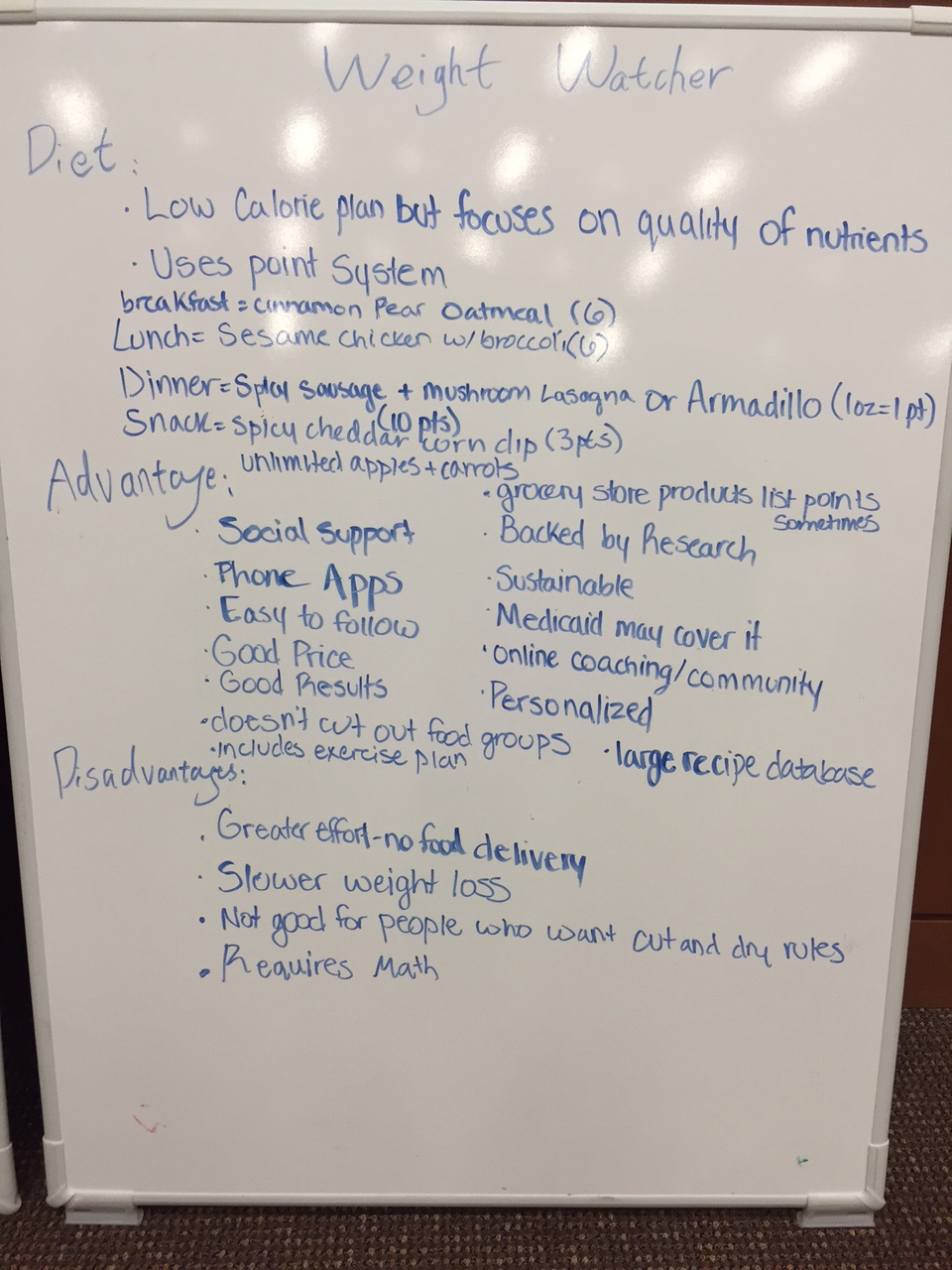


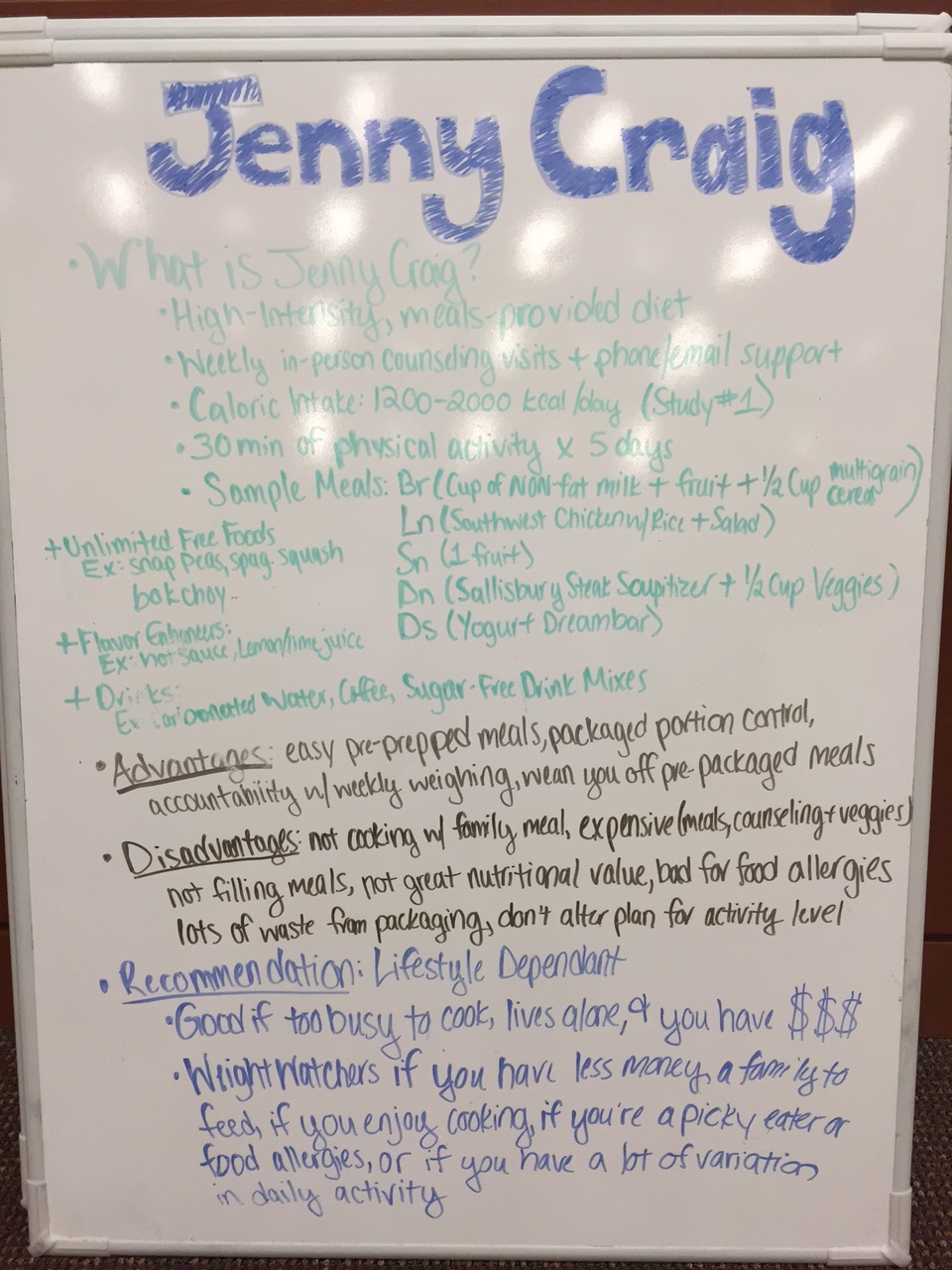


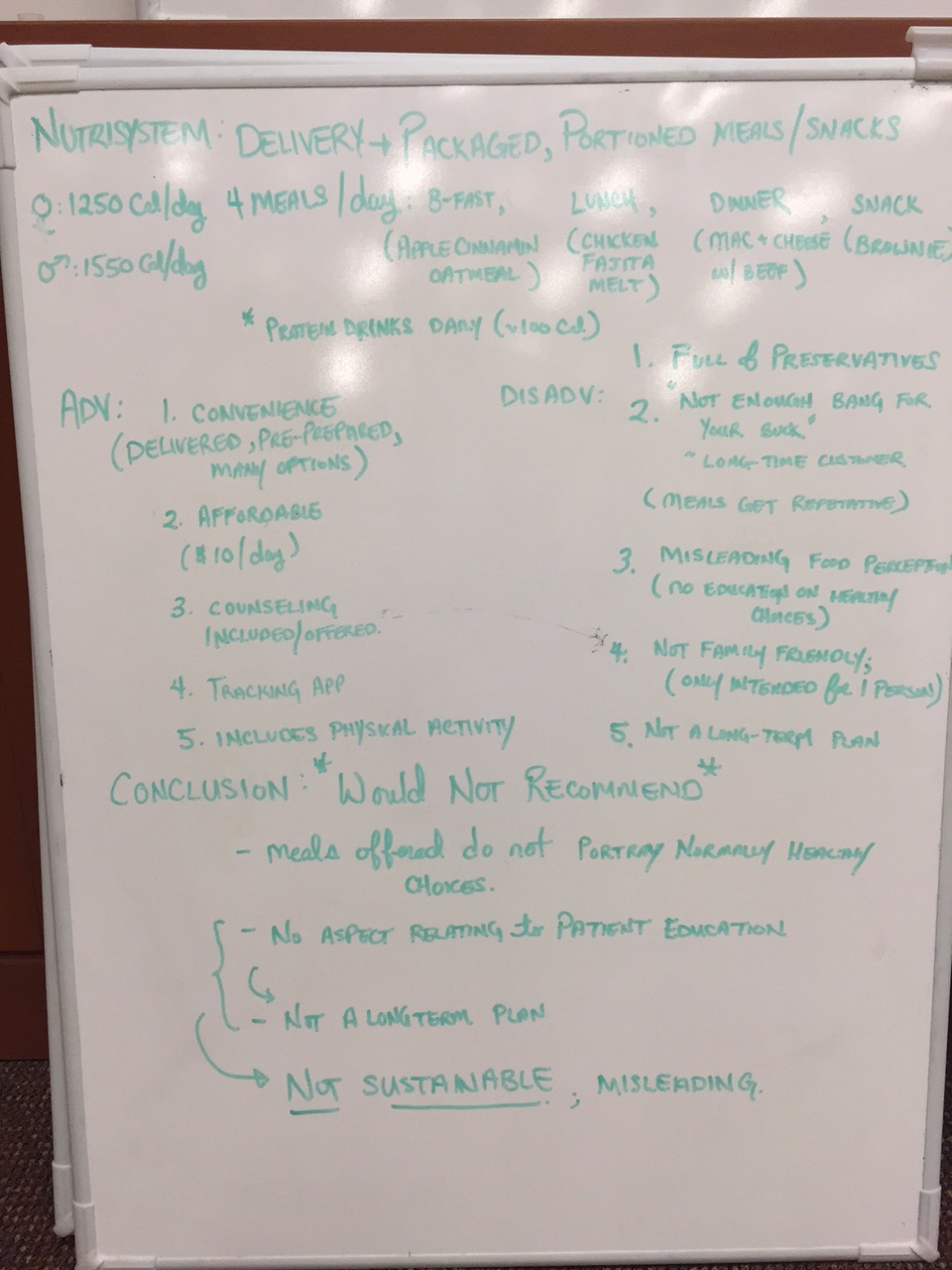


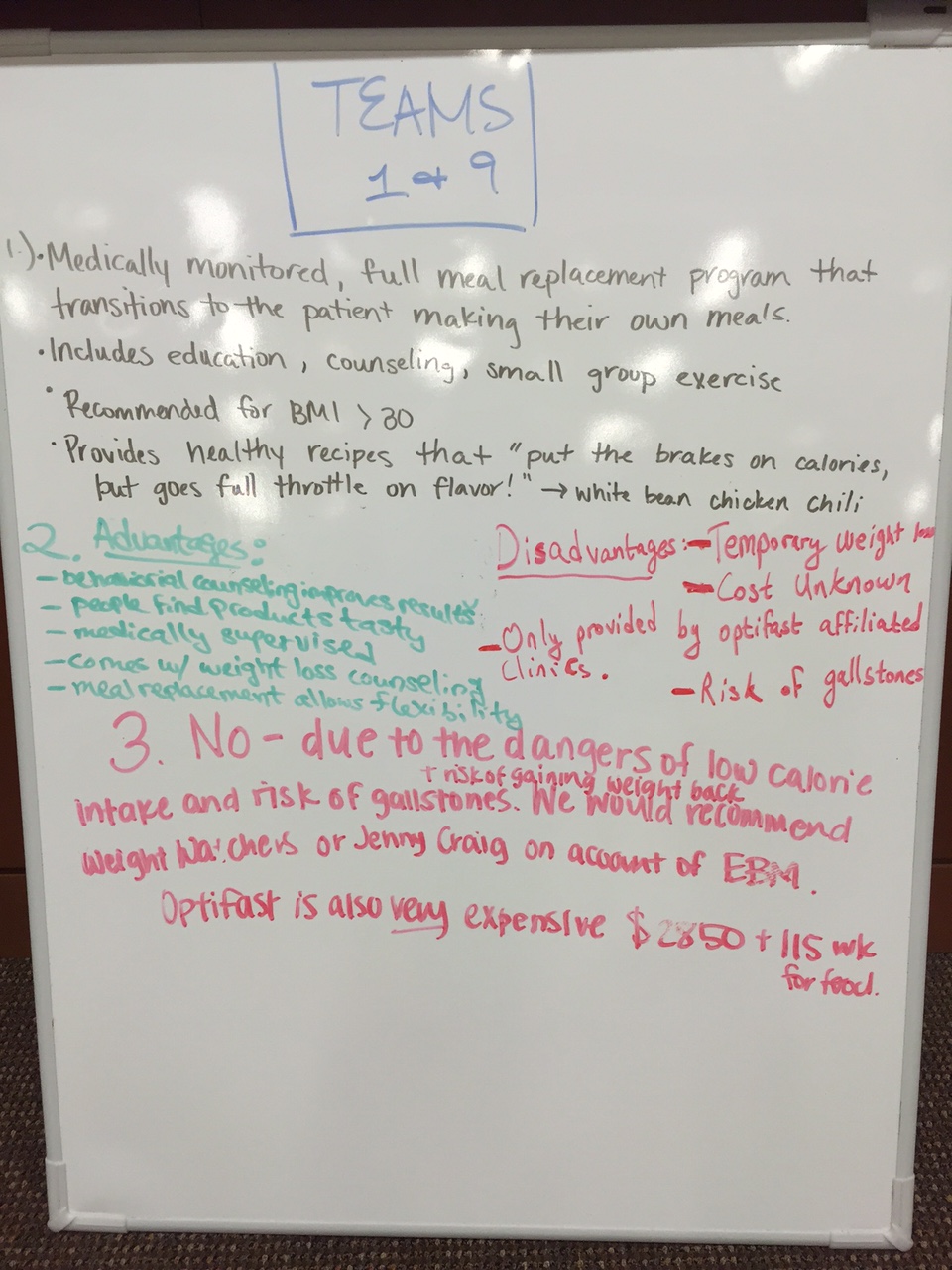


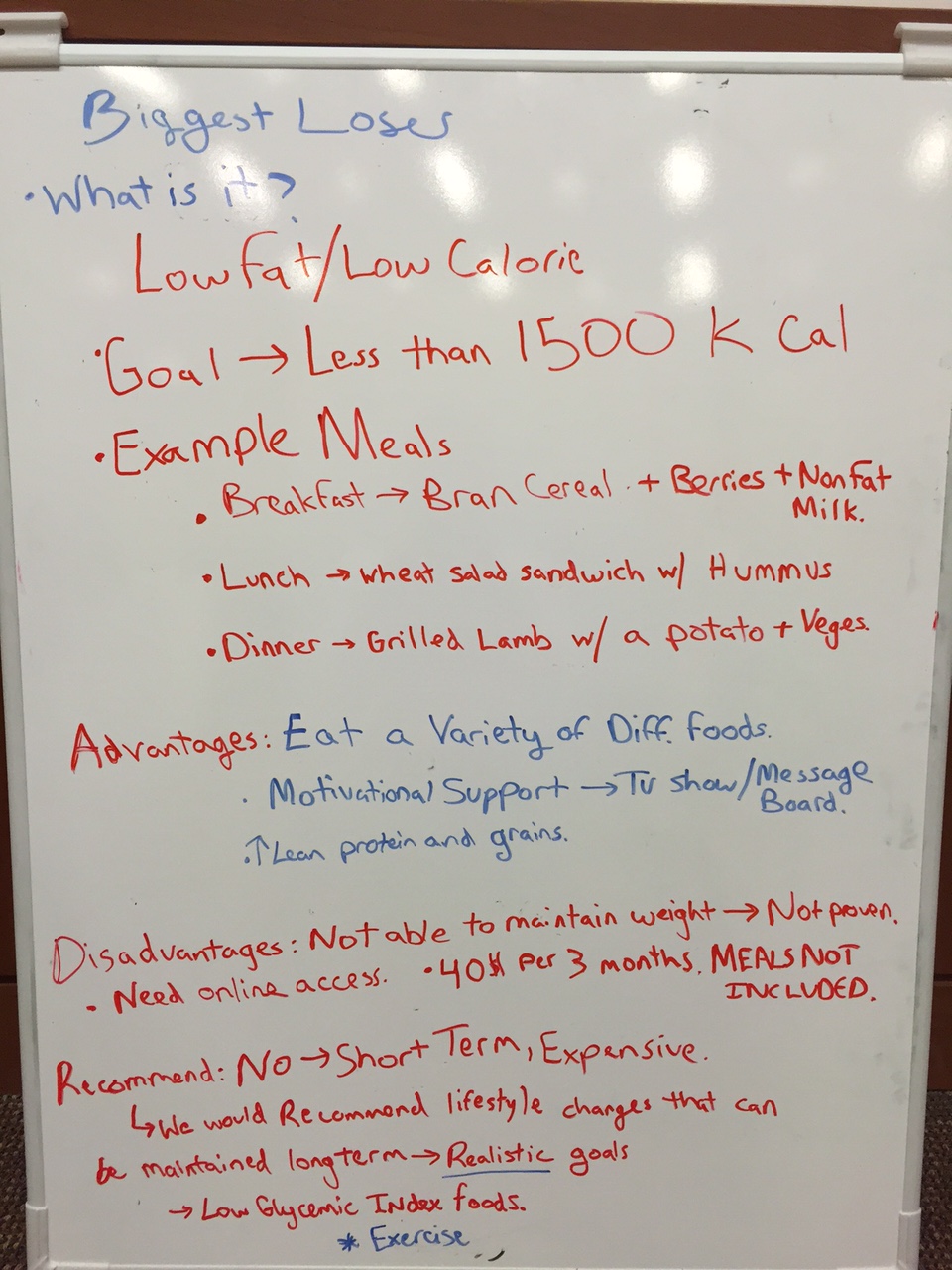


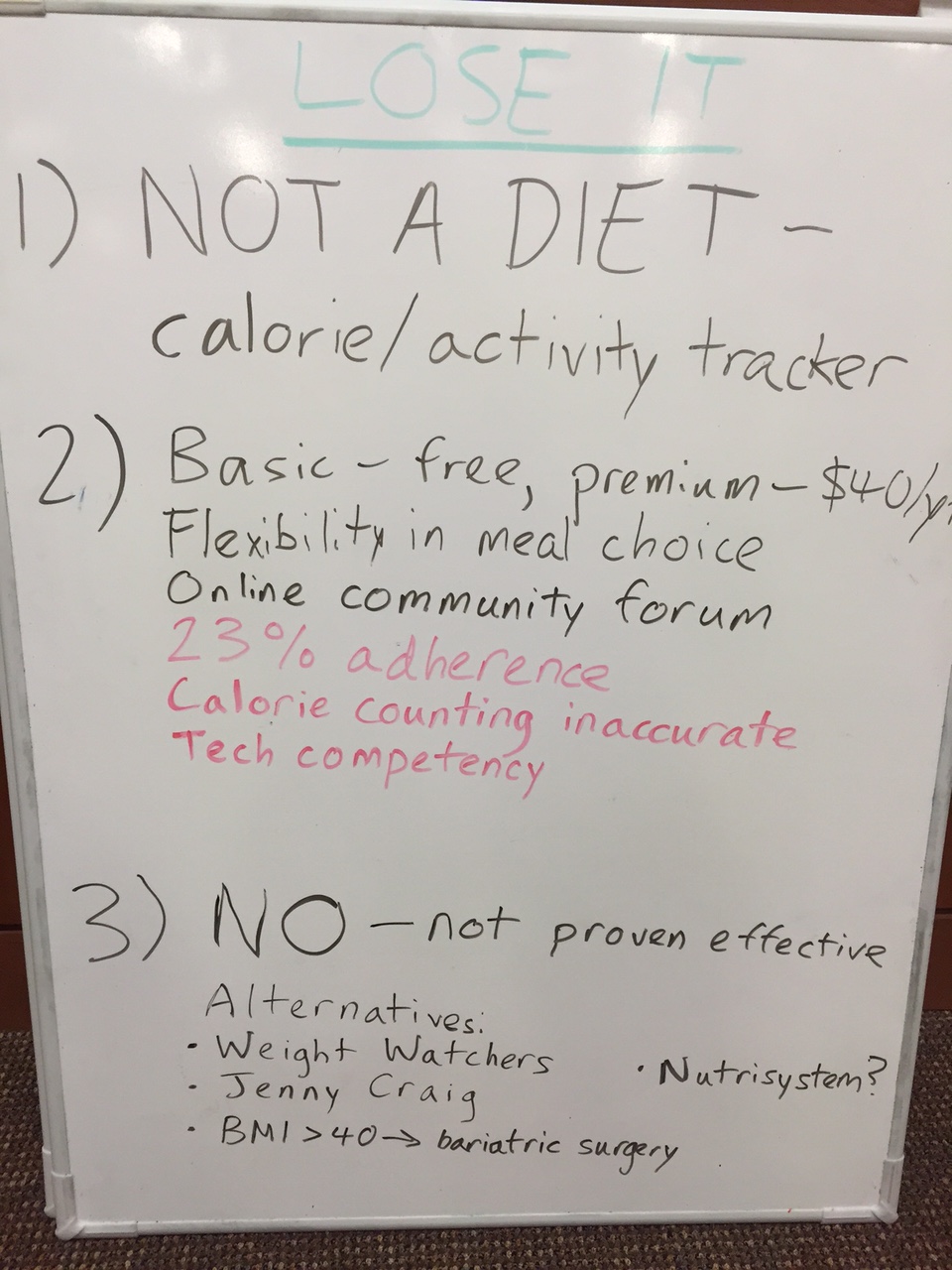


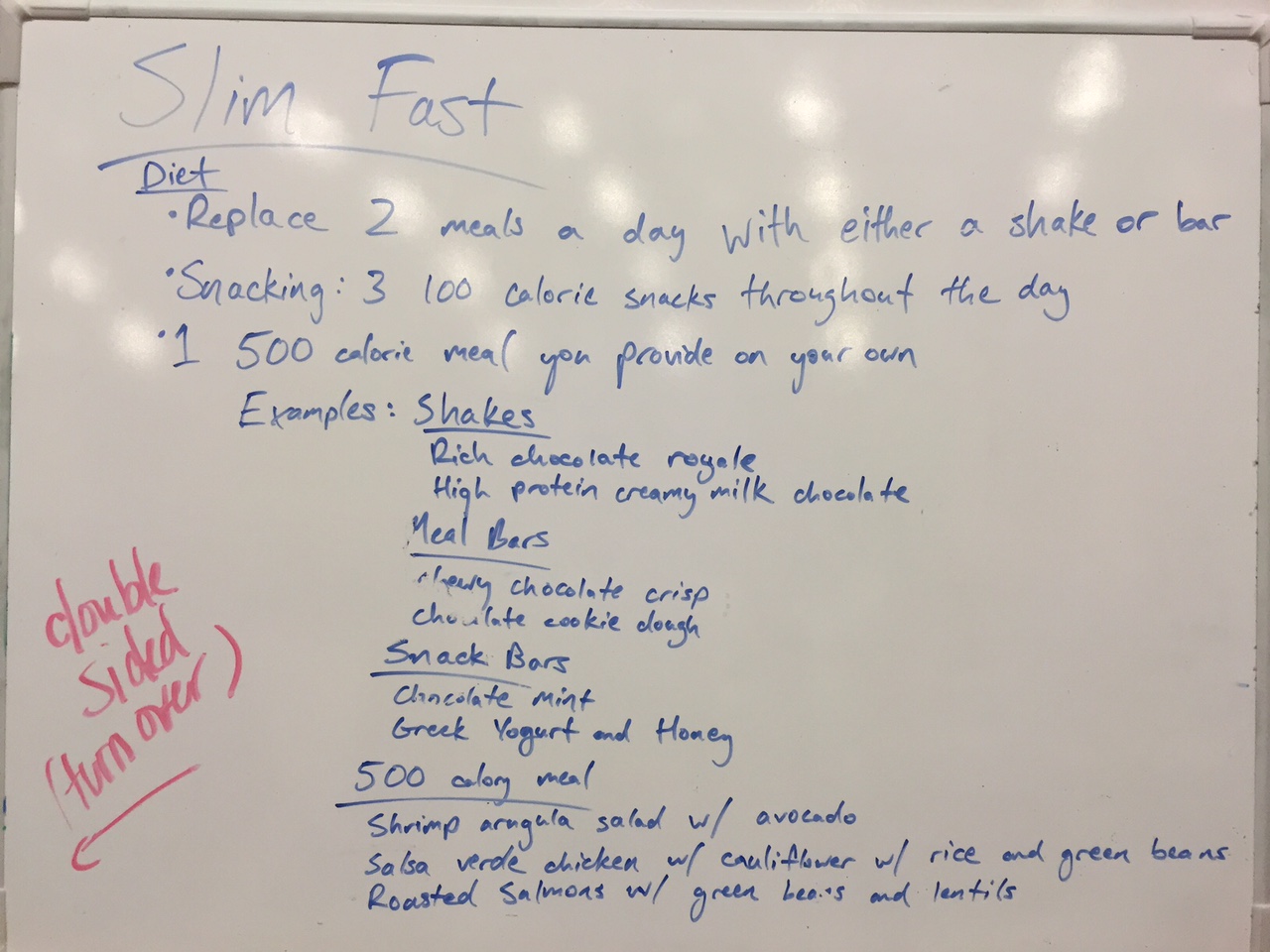


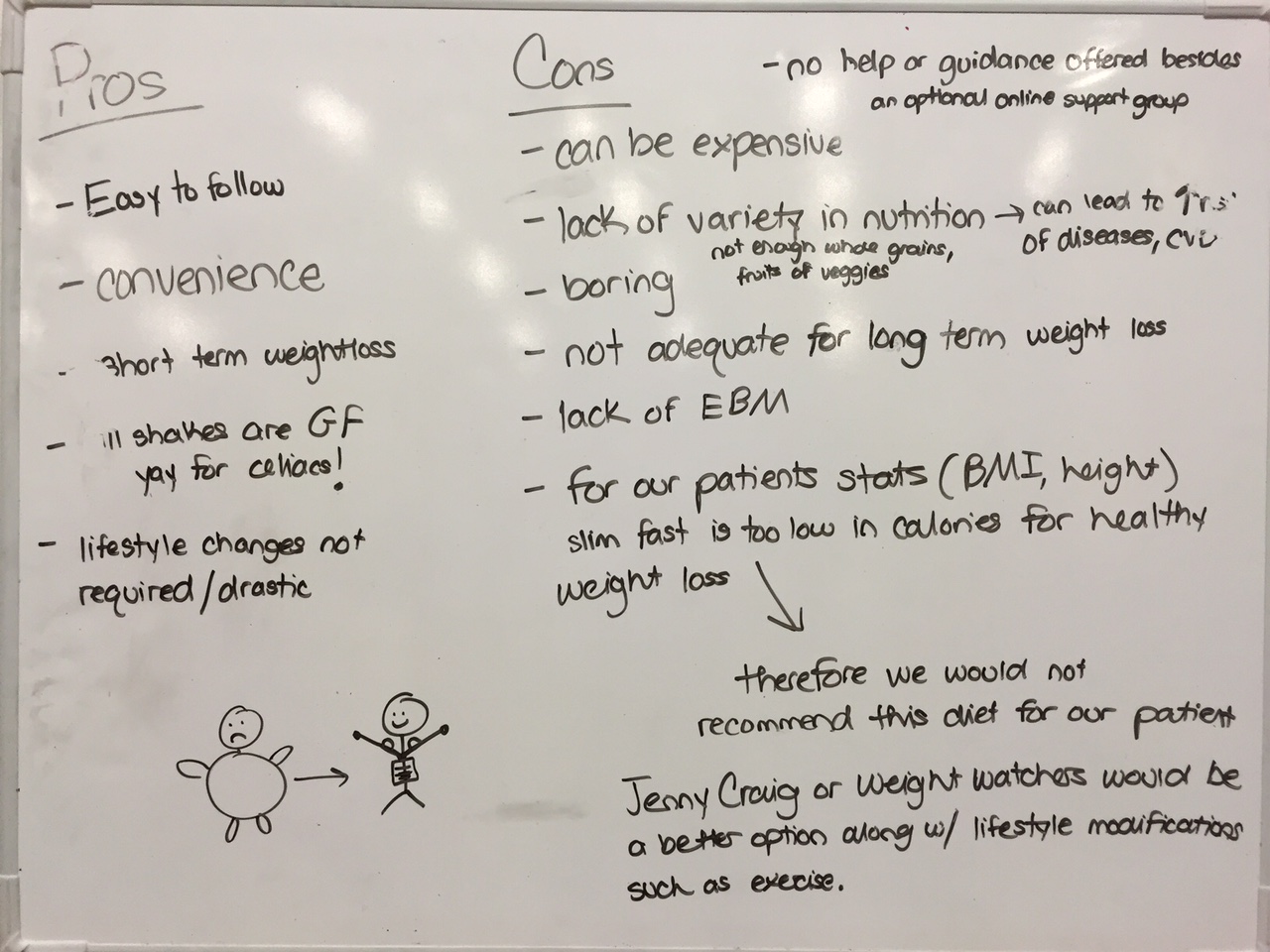

Supplement: Supplementary file 1 — A. Student Presentation.pptx B. Facilitators Guide.docx [file mep-12-10426-s001.zip › B. Facilitators Guide.docx]
