# Supplementary material for: Collaborative Learning Activity Utilizing Evidence-Based Medicine to Improve Medical Student Learning of the Lifestyle Management of Obesity
Source: MedEdPORTAL. 2016 Jul 21;12:10426. doi: 10.15766/mep_2374-8265.10426 (PMC6464419; doi:10.15766/mep_2374-8265.10426)
Supplement: Supplementary file 1 — A. Student Presentation.pptx B. Facilitators Guide.docx [file mep-12-10426-s001.zip › A. Student Presentation.pptx]

## Slide 1
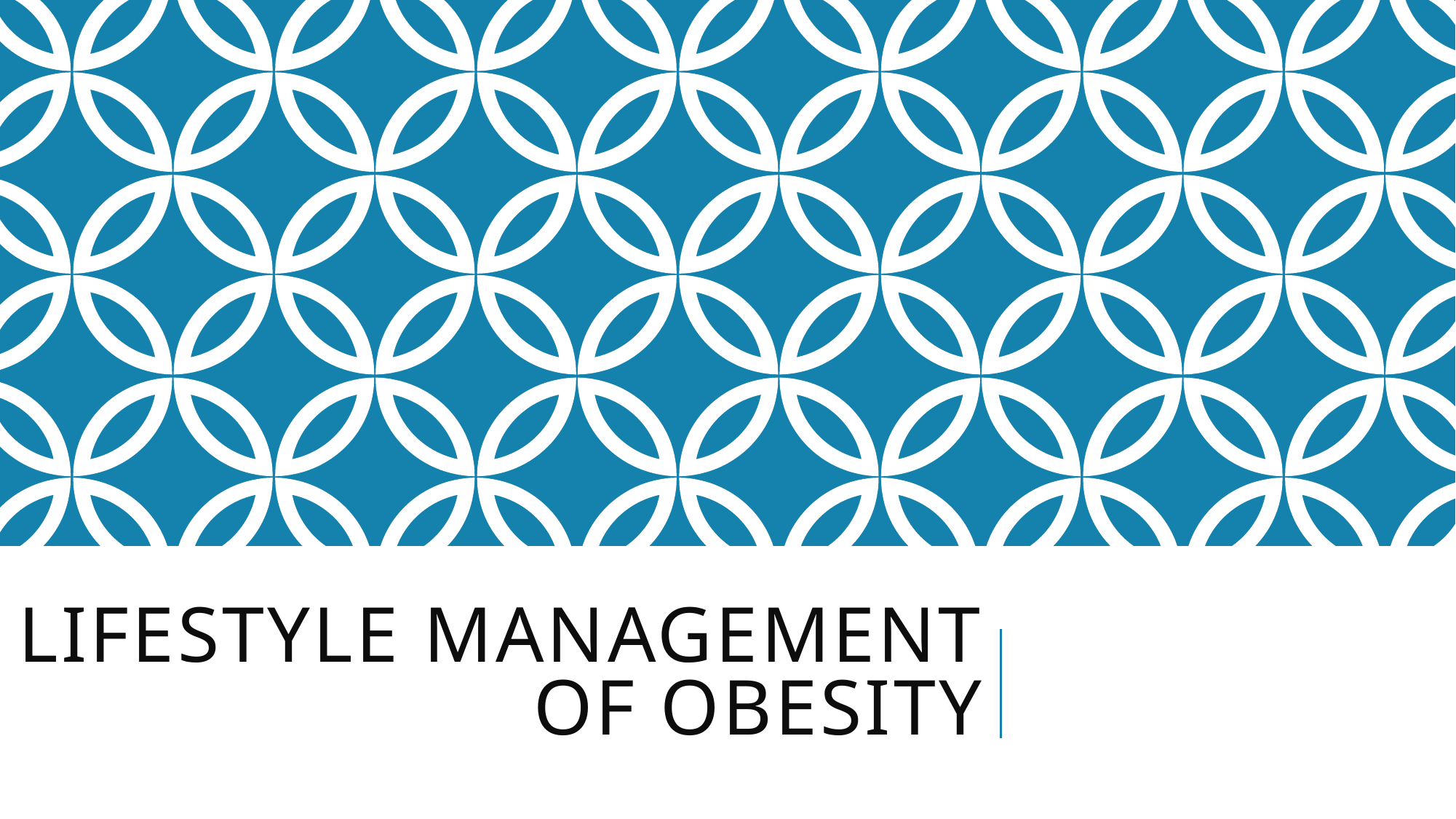

# Lifestyle management of obesity

## Slide 2
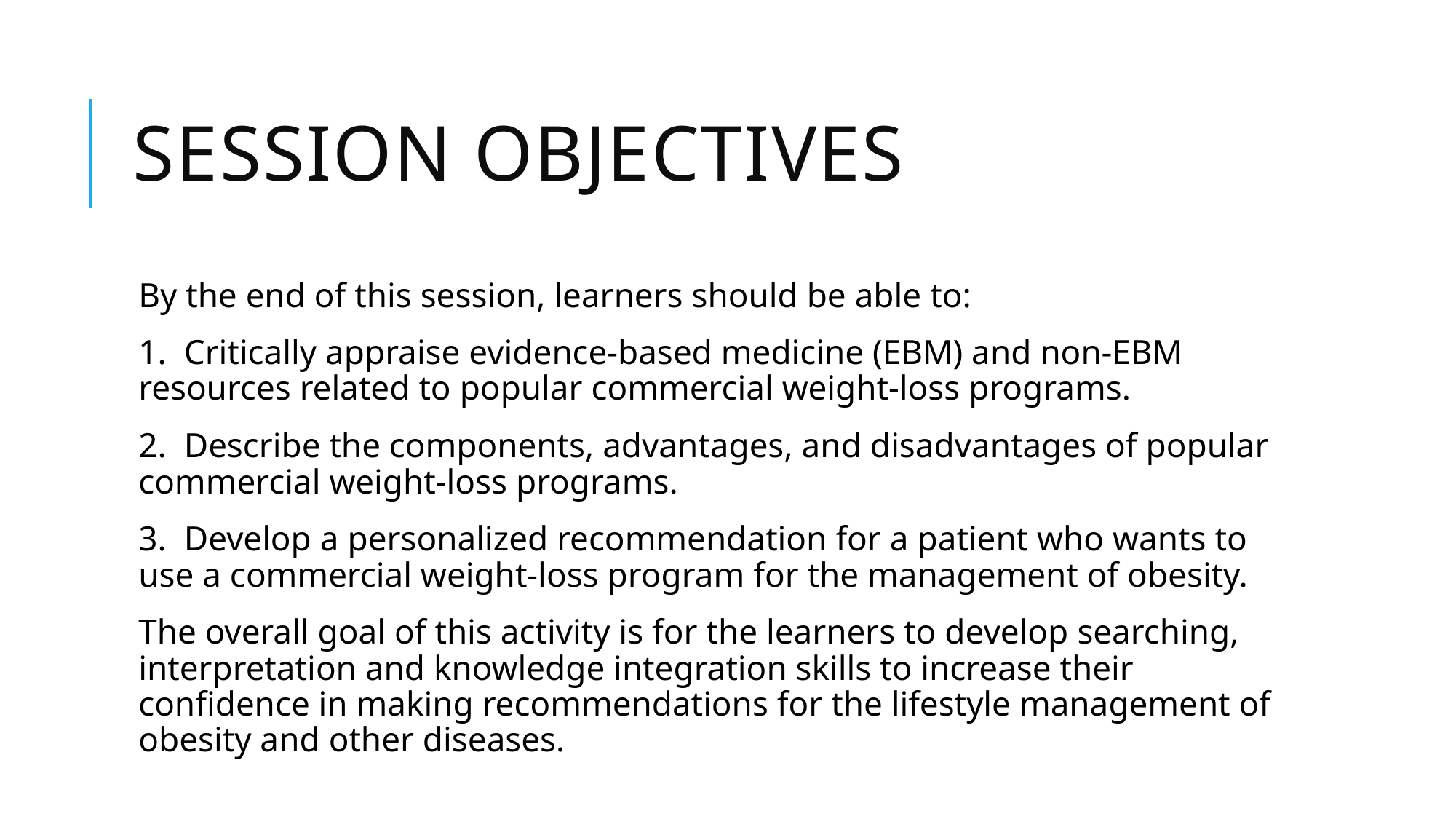

# Session objectives
By the end of this session, learners should be able to:
1.  Critically appraise evidence-based medicine (EBM) and non‐EBM resources related to popular commercial weight-loss programs.
2.  Describe the components, advantages, and disadvantages of popular commercial weight-loss programs.
3.  Develop a personalized recommendation for a patient who wants to use a commercial weight-loss program for the management of obesity.
The overall goal of this activity is for the learners to develop searching, interpretation and knowledge integration skills to increase their confidence in making recommendations for the lifestyle management of obesity and other diseases.

## Slide 3
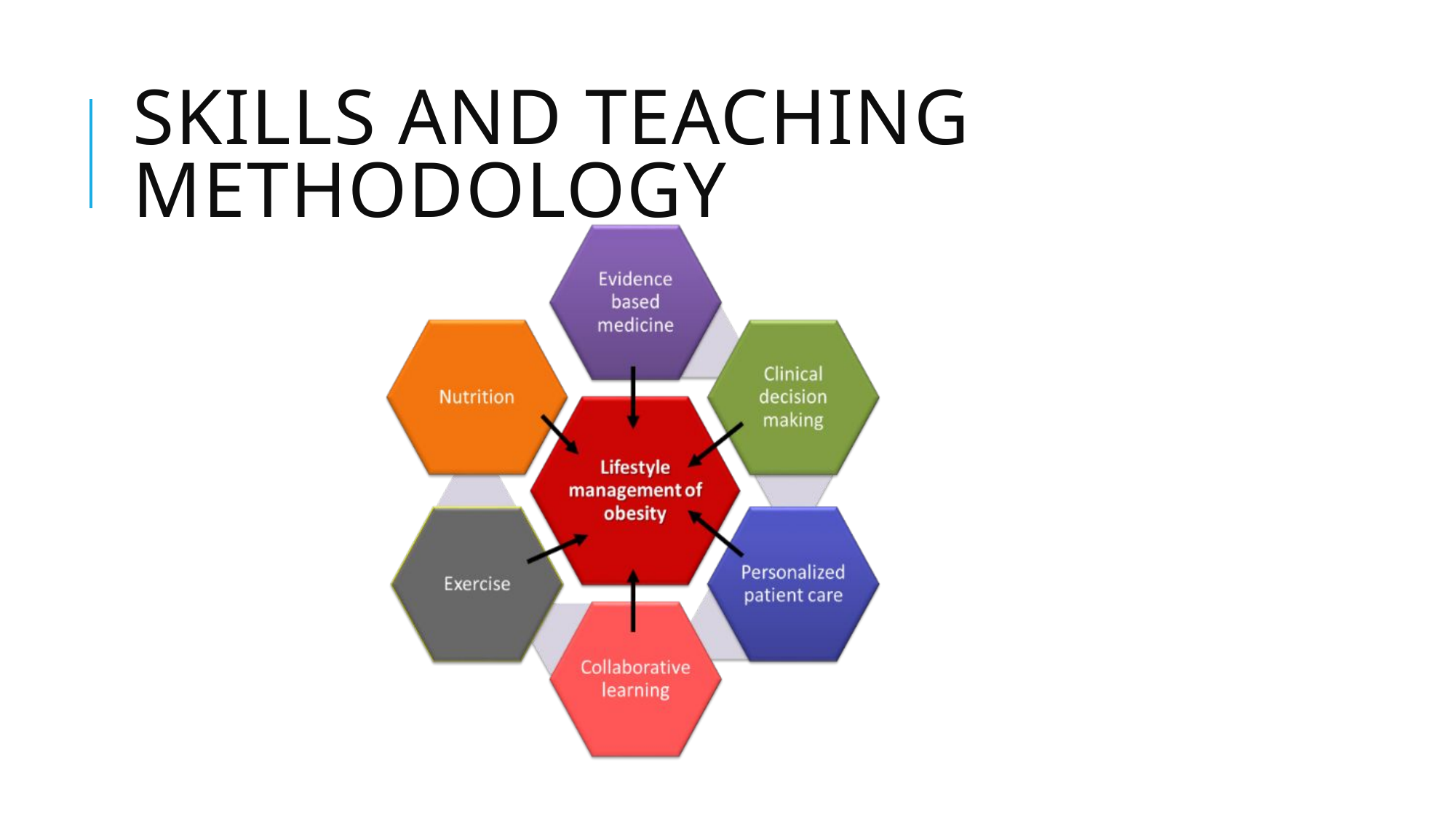

# Skills and teaching methodology

## Slide 4
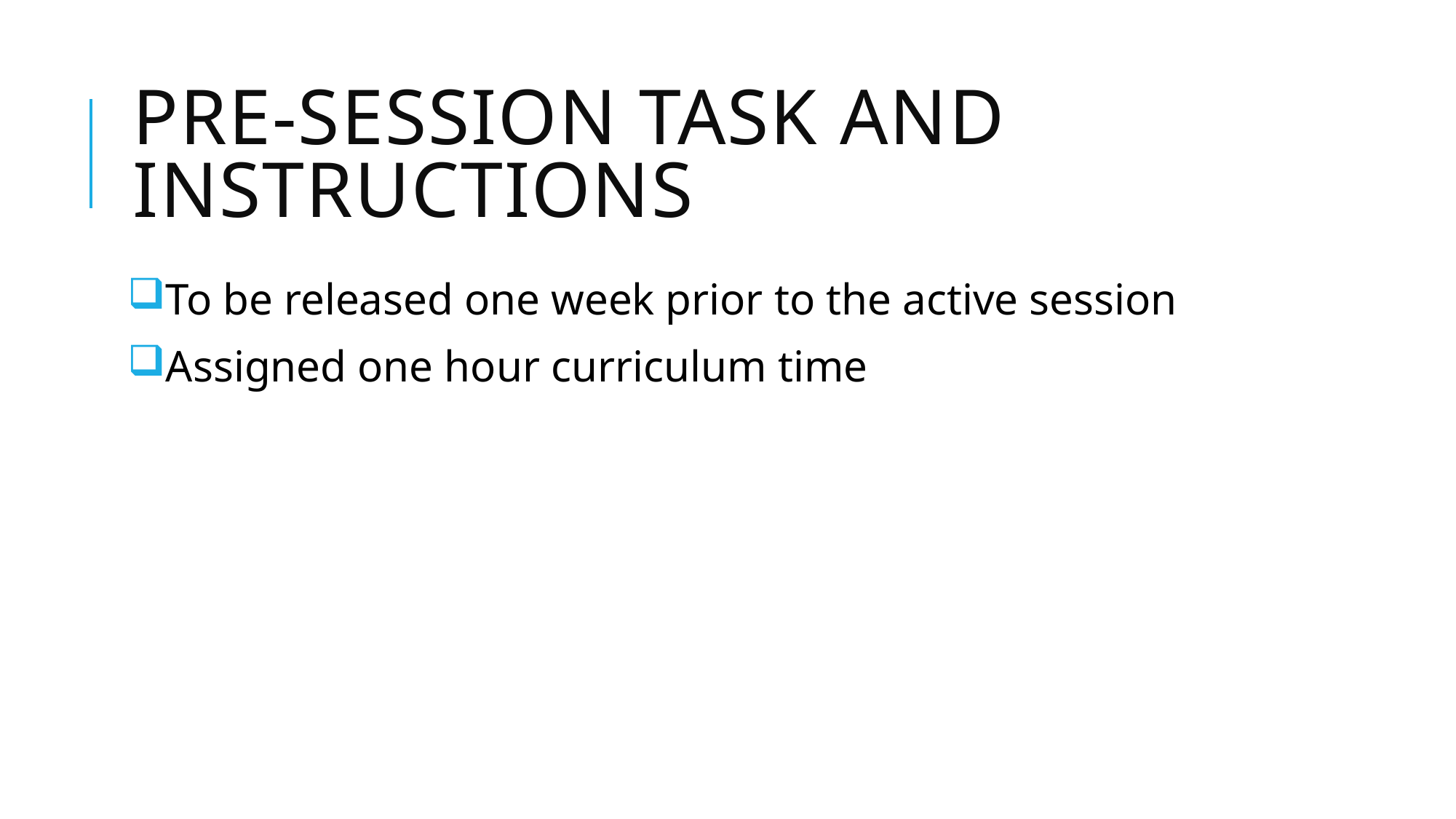

# Pre-session task and instructions
To be released one week prior to the active session
Assigned one hour curriculum time

## Slide 5
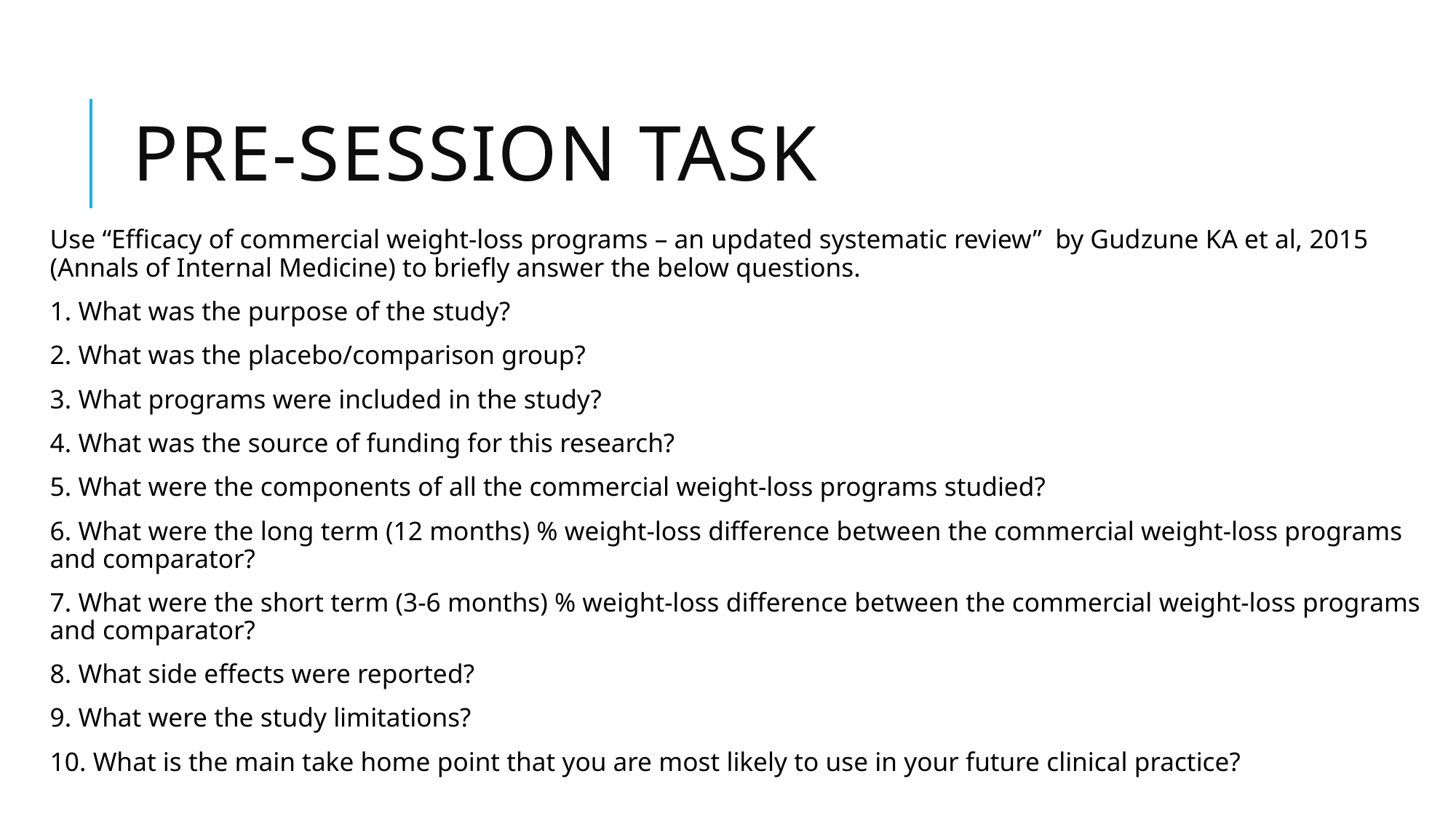

# Pre‐session task
Use “Efficacy of commercial weight‐loss programs – an updated systematic review”  by Gudzune KA et al, 2015 (Annals of Internal Medicine) to briefly answer the below questions.
1. What was the purpose of the study?
2. What was the placebo/comparison group?
3. What programs were included in the study?
4. What was the source of funding for this research?
5. What were the components of all the commercial weight-loss programs studied?
6. What were the long term (12 months) % weight-loss difference between the commercial weight-loss programs and comparator?
7. What were the short term (3‐6 months) % weight-loss difference between the commercial weight-loss programs and comparator?
8. What side effects were reported?
9. What were the study limitations?
10. What is the main take home point that you are most likely to use in your future clinical practice?

## Slide 6
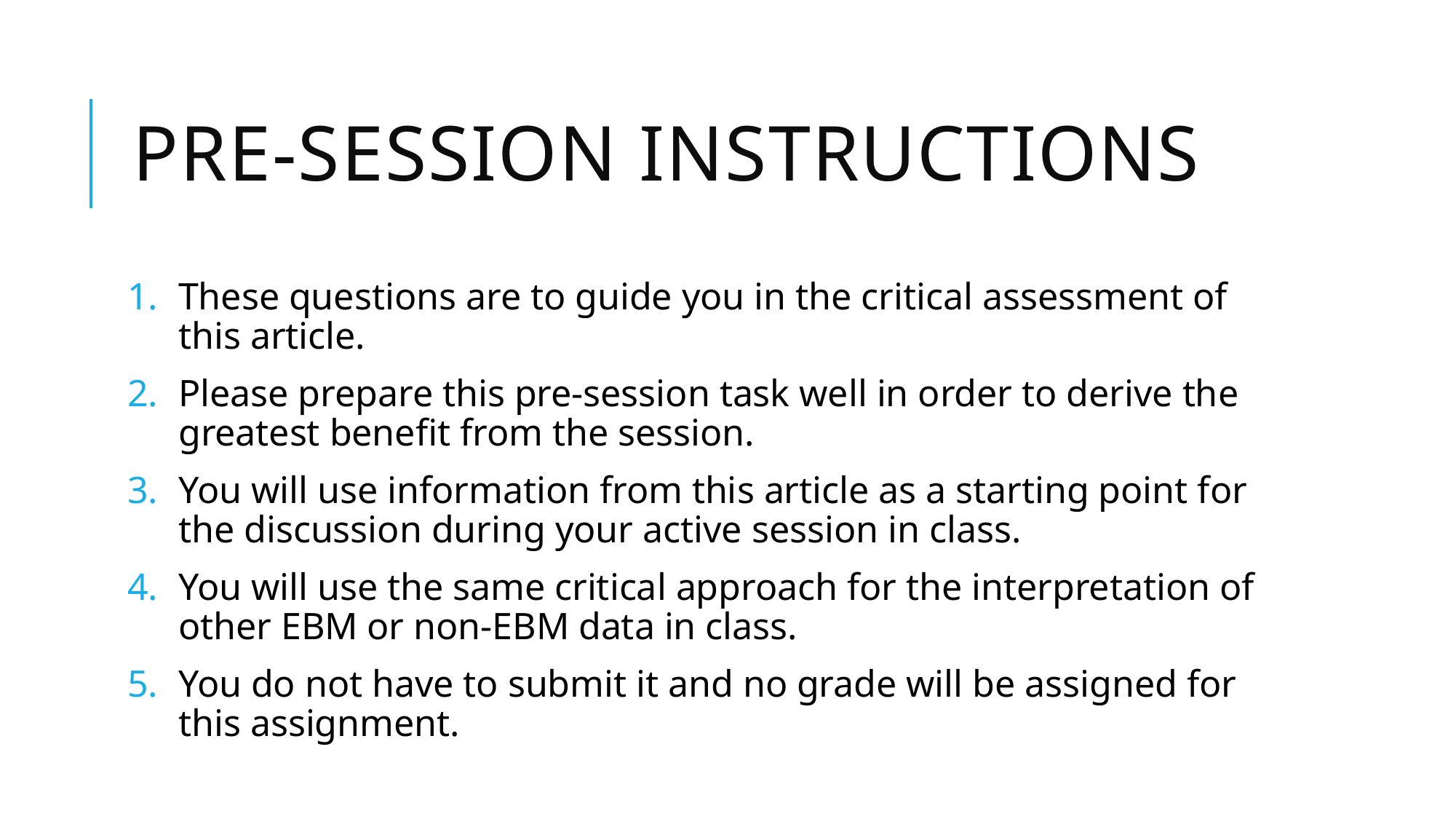

# Pre‐session instructions
These questions are to guide you in the critical assessment of this article.
Please prepare this pre-session task well in order to derive the greatest benefit from the session.
You will use information from this article as a starting point for the discussion during your active session in class.
You will use the same critical approach for the interpretation of other EBM or non‐EBM data in class.
You do not have to submit it and no grade will be assigned for this assignment.

## Slide 7
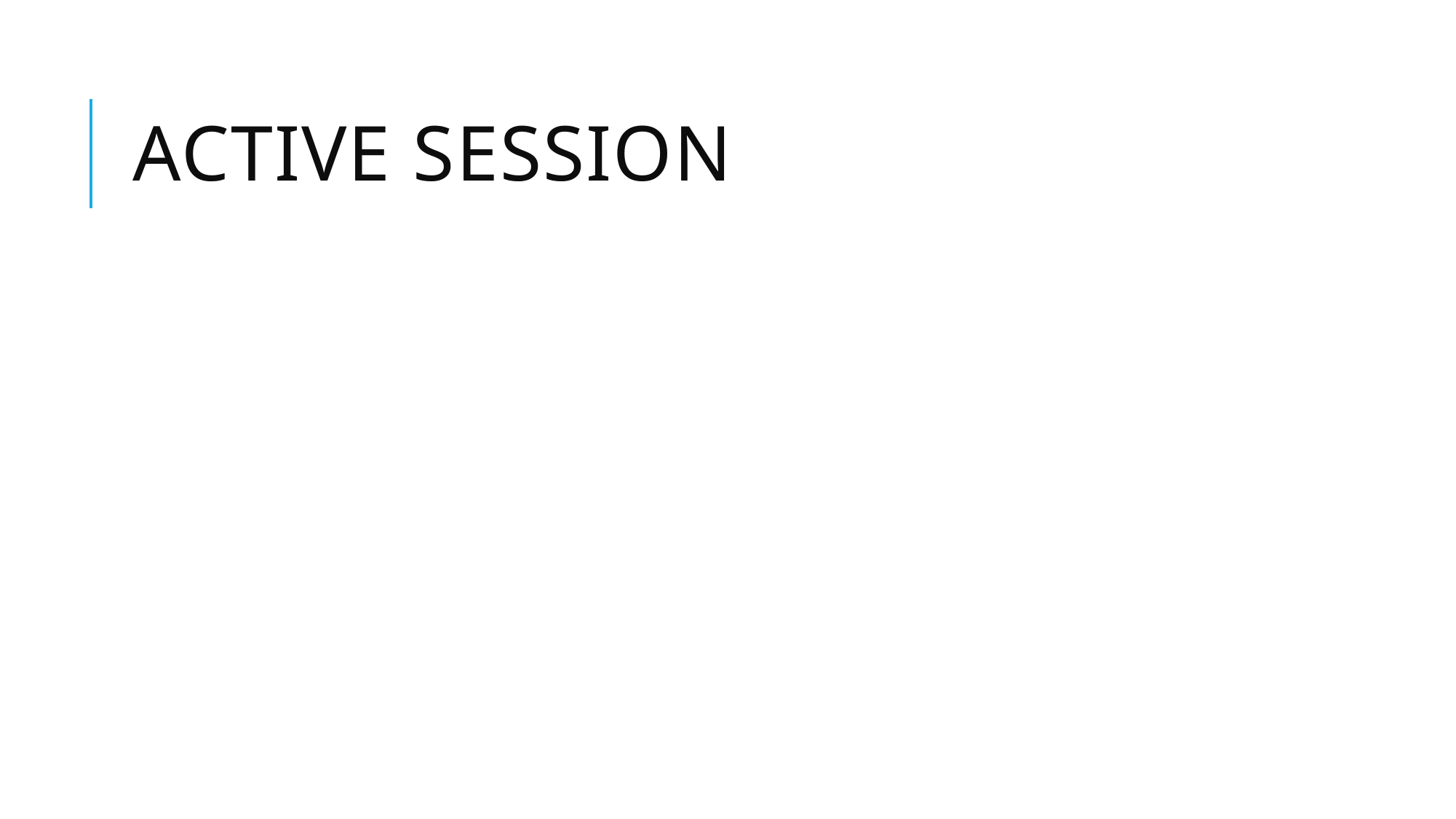

# Active session

## Slide 8
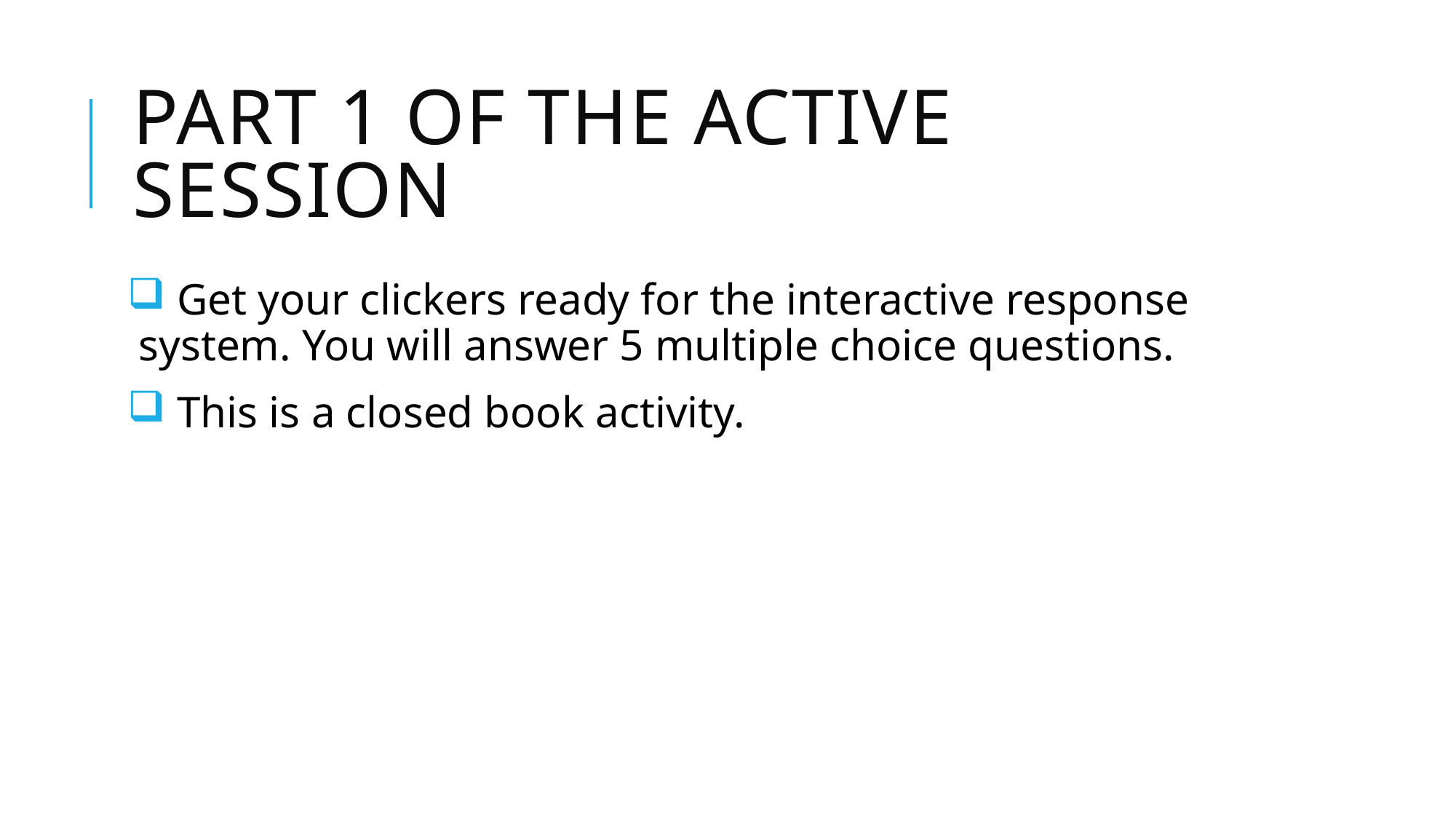

# Part 1 of the active session
 Get your clickers ready for the interactive response system. You will answer 5 multiple choice questions.
 This is a closed book activity.

## Slide 9
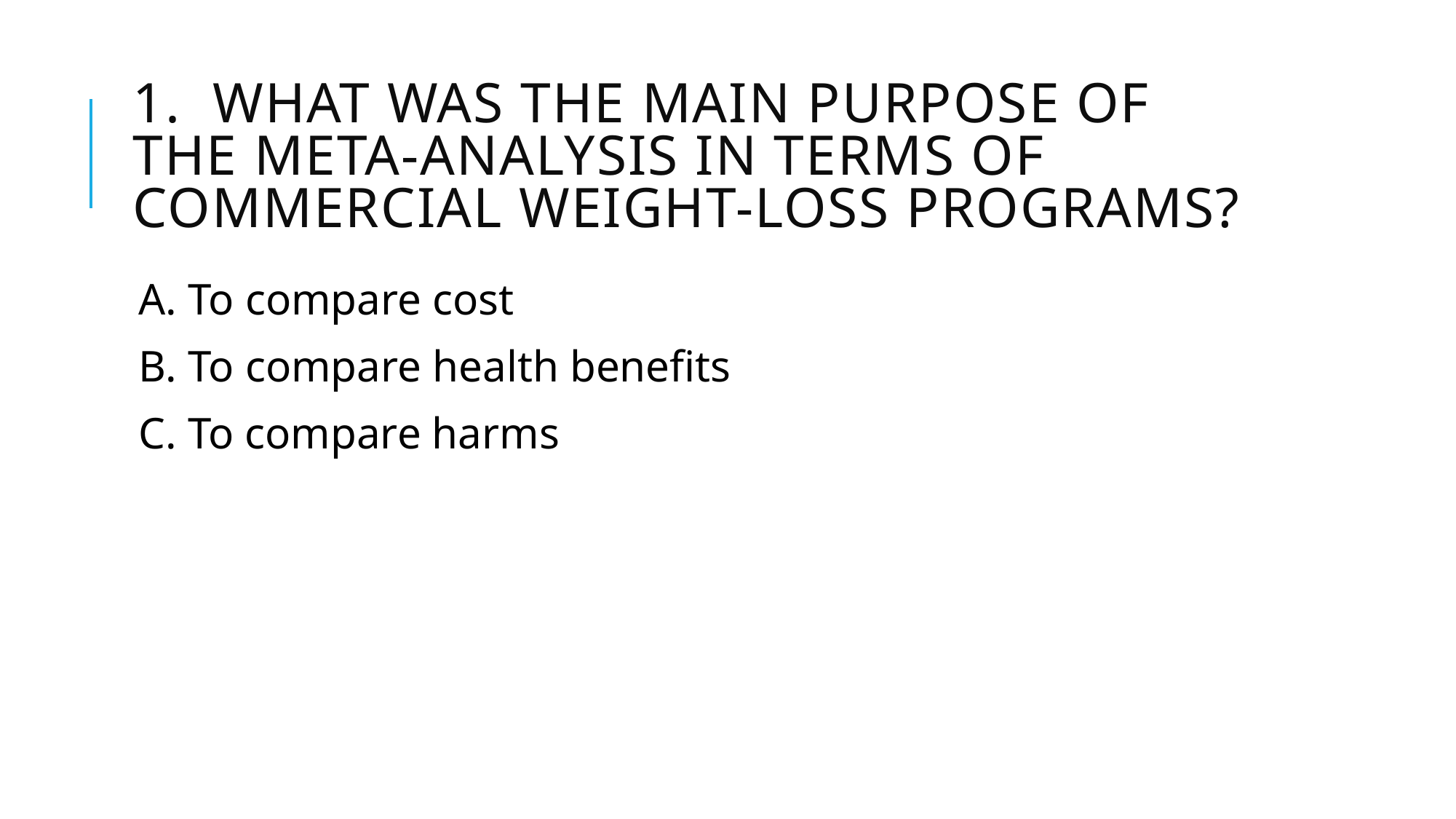

# 1.  What was the main purpose of the meta‐analysis in terms of commercial weight-loss programs?
A. To compare cost
B. To compare health benefits
C. To compare harms

## Slide 10
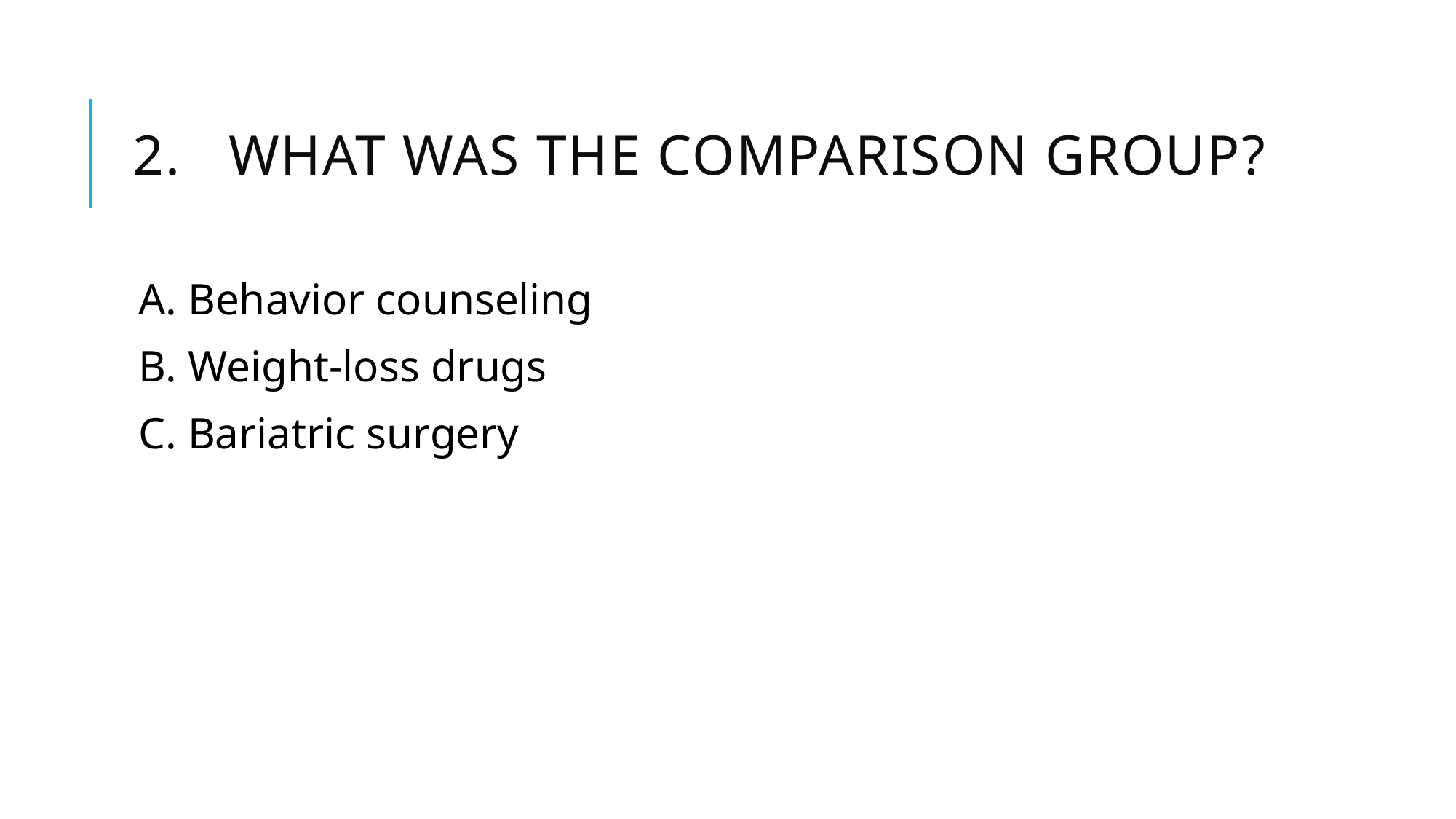

# 2.   What was the comparison group?
A. Behavior counseling
B. Weight-loss drugs
C. Bariatric surgery

## Slide 11
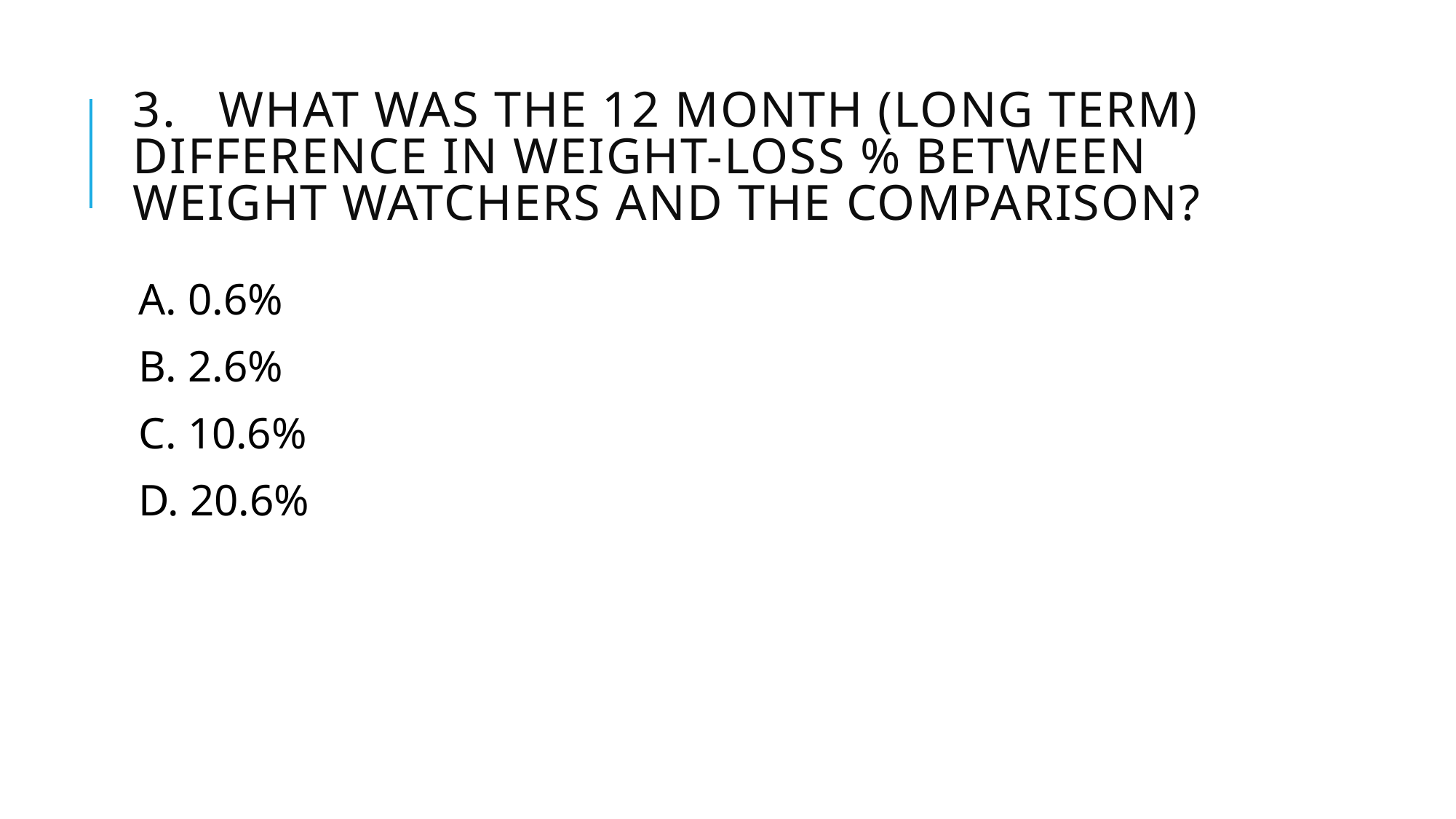

# 3.   What was the 12 month (long term) difference in weight-loss % between weight watchers and the comparison?
A. 0.6%
B. 2.6%
C. 10.6%
D. 20.6%

## Slide 12
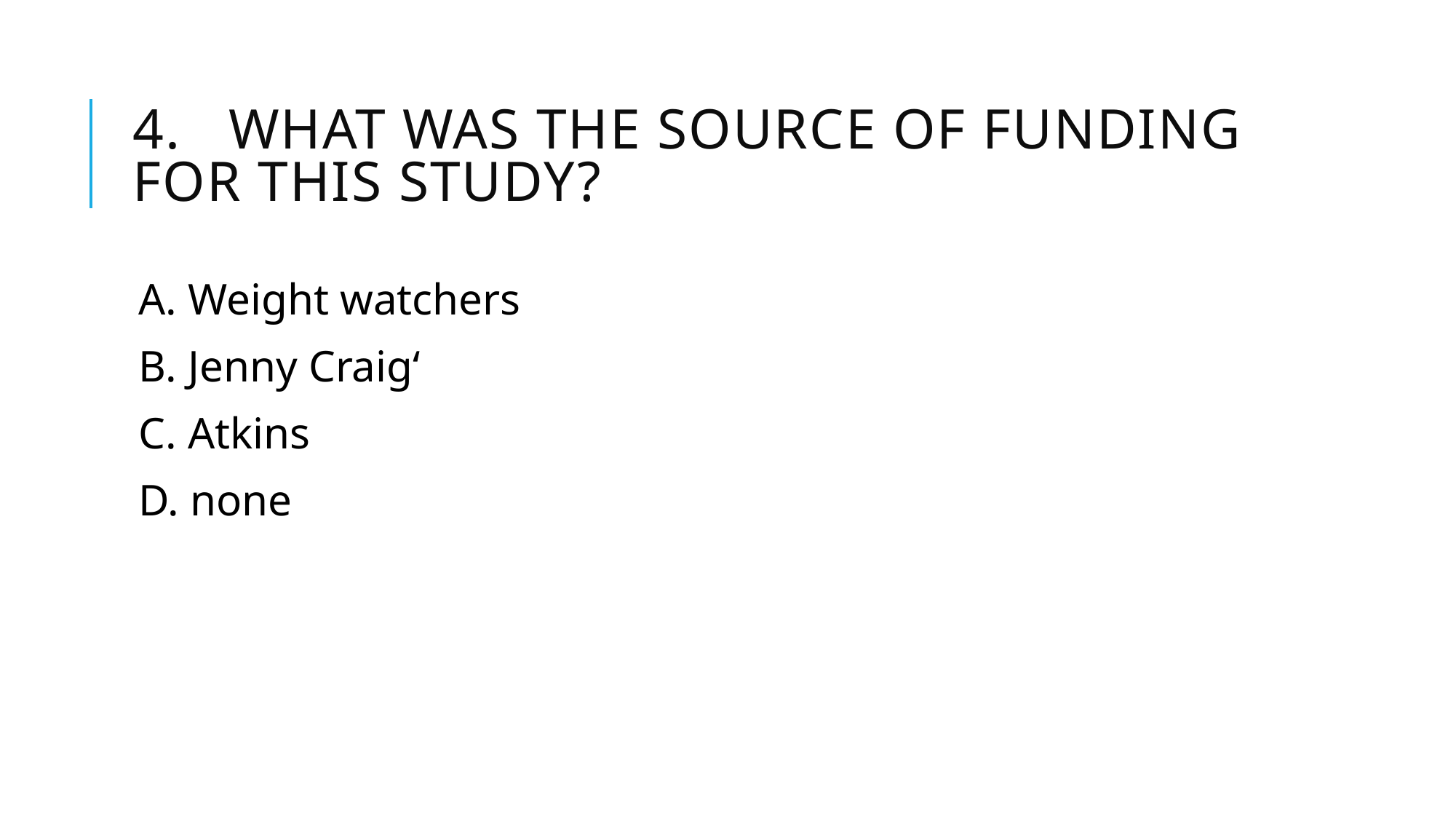

# 4.   What was the source of funding for this study?
A. Weight watchers
B. Jenny Craig‘
C. Atkins
D. none

## Slide 13
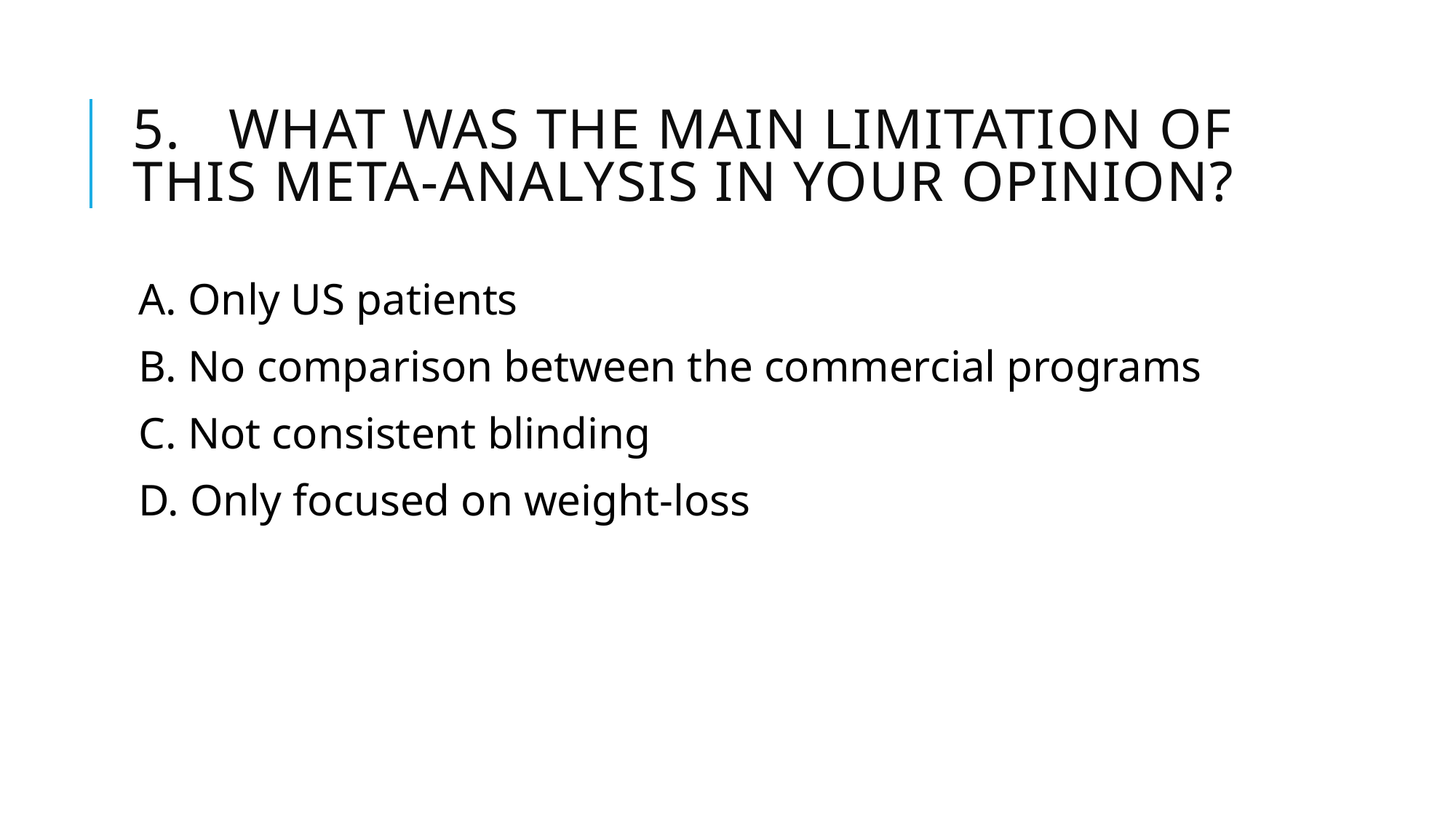

# 5.   What was the main limitation of this meta‐analysis in your opinion?
A. Only US patients
B. No comparison between the commercial programs
C. Not consistent blinding
D. Only focused on weight-loss

## Slide 14
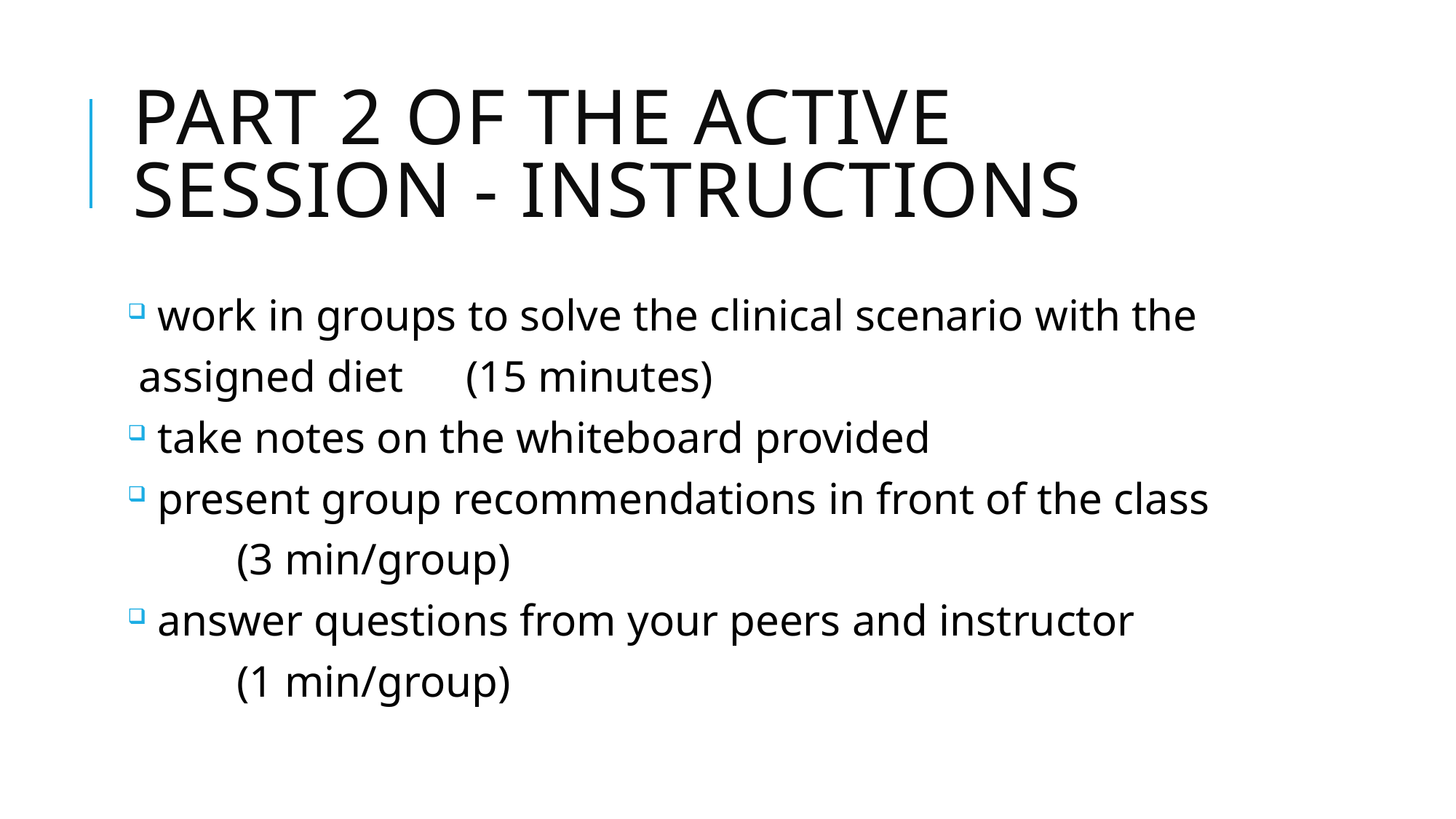

# Part 2 of the active session - instructions
 work in groups to solve the clinical scenario with the assigned diet 	(15 minutes)
 take notes on the whiteboard provided
 present group recommendations in front of the class
	(3 min/group)
 answer questions from your peers and instructor
	(1 min/group)

## Slide 15
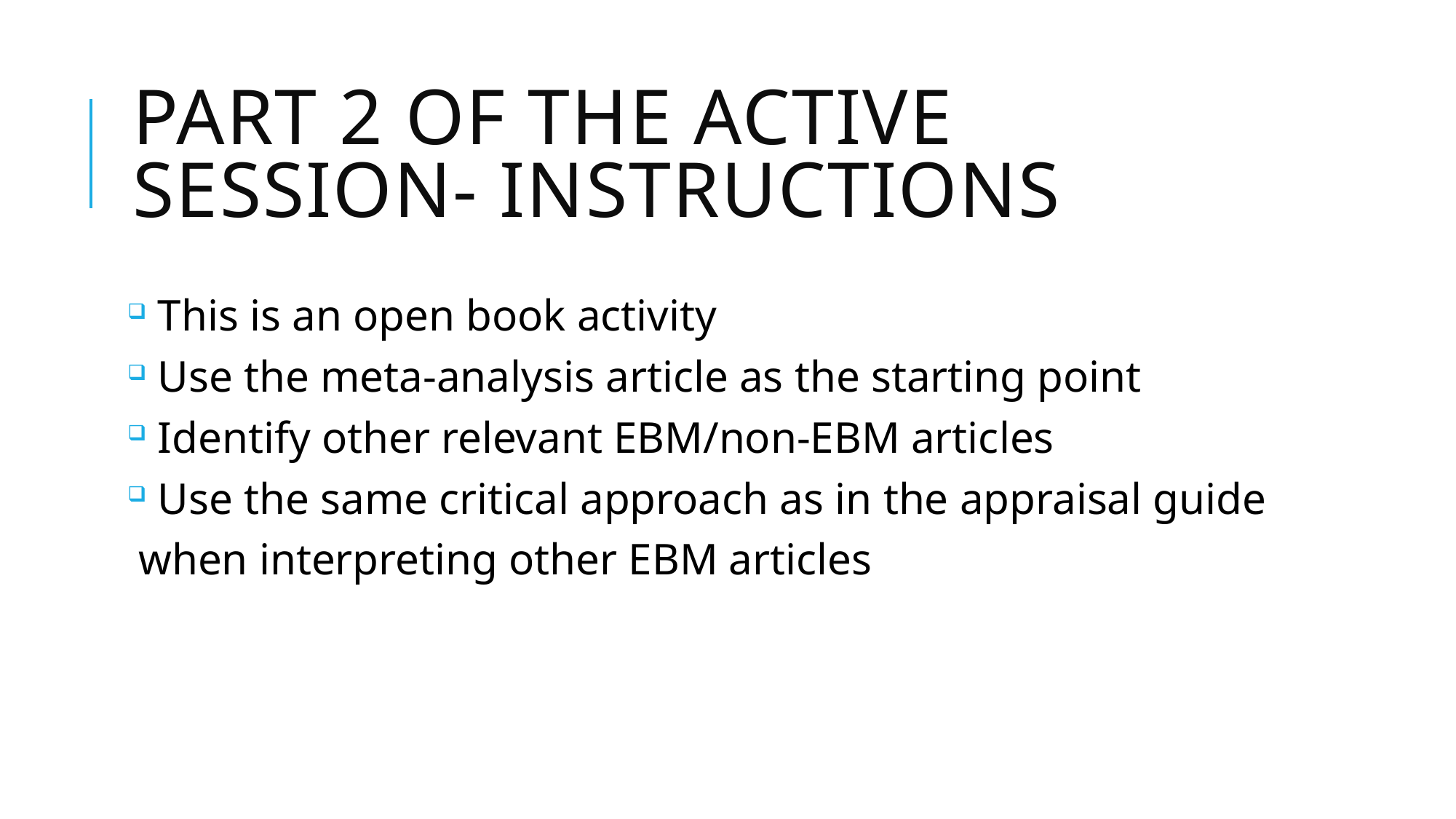

# Part 2 of the active session- instructions
 This is an open book activity
 Use the meta‐analysis article as the starting point
 Identify other relevant EBM/non‐EBM articles
 Use the same critical approach as in the appraisal guide when interpreting other EBM articles

## Slide 16
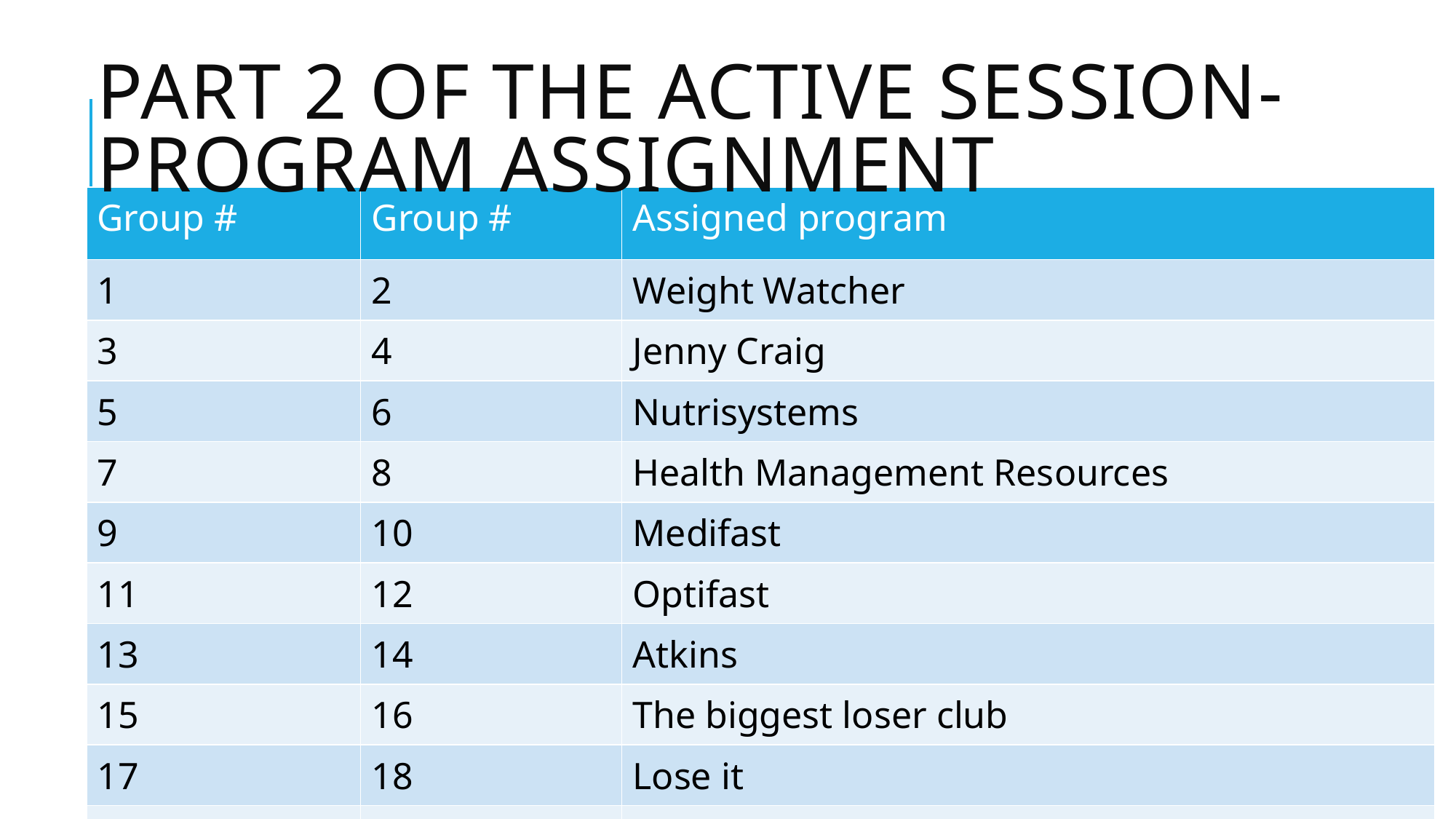

# Part 2 of the active session- Program assignment
| Group # | Group # | Assigned program |
| --- | --- | --- |
| 1 | 2 | Weight Watcher |
| 3 | 4 | Jenny Craig |
| 5 | 6 | Nutrisystems |
| 7 | 8 | Health Management Resources |
| 9 | 10 | Medifast |
| 11 | 12 | Optifast |
| 13 | 14 | Atkins |
| 15 | 16 | The biggest loser club |
| 17 | 18 | Lose it |
| 19 | 20 | Slim fast |

## Slide 17
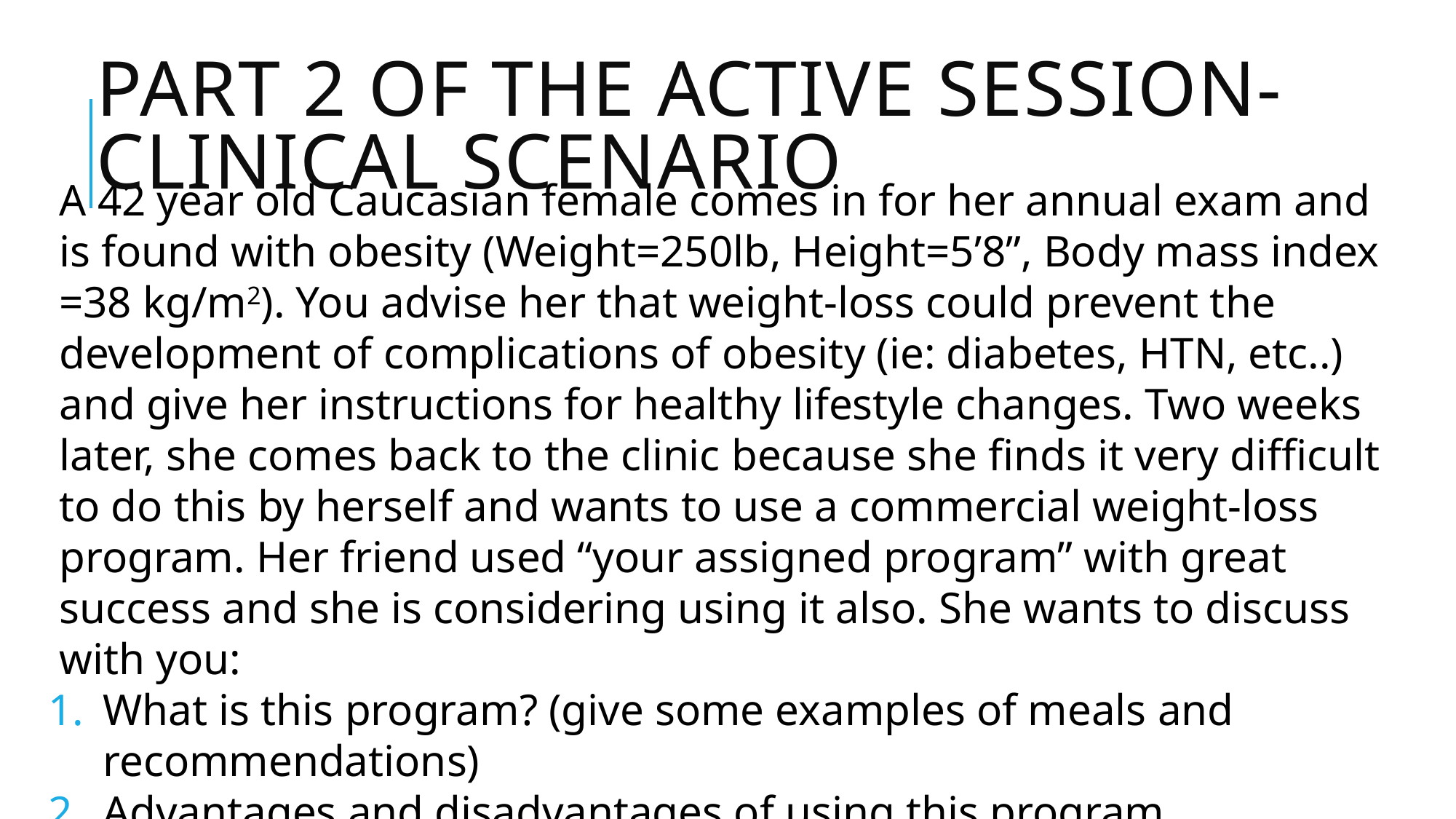

Part 2 of the active session- clinical scenario
A 42 year old Caucasian female comes in for her annual exam and is found with obesity (Weight=250lb, Height=5’8”, Body mass index =38 kg/m2​​). You advise her that weight-loss could prevent the development of complications of obesity (ie: diabetes, HTN, etc..) and give her instructions for healthy lifestyle changes. Two weeks later, she comes back to the clinic because she finds it very difficult to do this by herself and wants to use a commercial weight-loss program. Her friend used “your assigned program” with great success and she is considering using it also. She wants to discuss with you:
What is this program? (give some examples of meals and recommendations)
Advantages and disadvantages of using this program
Would you recommend her to use this program? If not, which other program would you recommend and why?
